# Diet alters performance and transcription patterns in *Oedaleus asiaticus* (Orthoptera: Acrididae) grasshoppers

**DOI:** 10.1371/journal.pone.0186397

**Published:** 2017-10-12

**Authors:** Xunbing Huang, Douglas W. Whitman, Jingchuan Ma, Mark Richard McNeill, Zehua Zhang

**Affiliations:** 1 State Key Laboratory of Biology of Plant Diseases and Insect Pests, Institute of Plant Protection, Chinese Academy of Agricultural Sciences, Beijing, P.R. China; 2 Scientific Observation and Experimental Station of Pests in Xilin Gol Rangeland, Institute of Plant Protection, Chinese Academy of Agricultural Sciences, Xilinhot, P.R. China; 3 School of Biological Sciences, Illinois State University, Normal, Illinois, United States of America; 4 AgResearch, Lincoln Research Centre, Christchurch, New Zealand; USDA Agricultural Research Service, UNITED STATES

## Abstract

We reared *Oedaleus asiaticus* grasshoppers under four different single-plant diets to examine the relationships among diet, performance, stress, and transcription patterns. Grasshoppers fed only *Artemisia frigida* (Asteraceae) were stressed, as indicated by their lower growth, size, development, and survival, in comparison to grasshoppers fed on any of three grasses, *Cleistogenes squarrosa*, *Leymus chinensis*, or *Stipa krylovii* (all Poaceae). We then used transcriptome analysis to examine how gene expression levels in *O*. *asiaticus* were altered by feeding on these diets. Nymphs fed *A*. *frigida* had the largest variation in gene expression profiles with a total of 299 genes significantly up- or down-regulated compared to those feeding on the three grasses: down-regulated genes included those involved in cuticle biosynthesis, DNA replication, biosynthesis and metabolism of nutrition. The up-regulated genes included stress-resistant and detoxifying enzymes. GO and KEGG enrichment analysis also showed that feeding on *A*. *frigida* could down-regulate biosynthesis and metabolism related pathways, and up-regulate stress-resistant and detoxification terms and pathways. Our results show that diet significantly altered gene-expression, and that unfavorable, stressful diets induce more transcriptional changes than favorable diets. Altered gene-expression represents phenotypic plasticity, and many such changes appear to be evolved, adaptive responses. The ease and regularity by which individuals shift phenotypes via altered transcription suggests that populations consist not of similar, fixed phenotypes, but of a collection of ever-changing, divergent phenotypes.

## Introduction

Phenotypic plasticity refers to the ability of individuals to alter their phenotypes in response to changing environments [1]. Phenotypic plasticity is of immense importance in biology, because it allows individuals to adapt in real time by altering their biochemistry, morphology, physiology, development, behavior, or life-history [1–3]. These changes can increase survival, fecundity, fitness, population density, and species range, and hence have both ecological and evolutionary consequences [1, 3].

An interesting and important category of phenotypic plasticity relates to how animals alter their phenotypes in response to different foods. Diet resources vary in space and time, and it is not surprising that animals have evolved the ability to rapidly change their phenotypes in response to these challenges. Many animals alter their biochemical phenotypes to better optimize favorable food items, or, conversely, to ameliorate unfavorable food items. An example of the former is the rapid production of lipases, carbohydrases, or proteases after consuming lipids, sugars, or proteins [4–6]. Examples of the latter include the rapid synthesis of mixed-function oxidases, after consuming toxic plants, or the generation of lipid-synthesizing enzymes in response to lipid-deficient diets [7–10]. These types of rapid biochemical response to changing nutrition are vitally important for animals [11–12], as shown by the fact that the loss of the ability to down- or up-regulate any one of hundreds of nutritional or detoxifying enzymes can be debilitating, as is seen in hyperlipemia individuals [13].

In addition to diet’s direct influence on nutritional biochemistry, altered diets also induce numerous broader, derivative (down-stream) changes to the phenotype, including to general homeostasis, physiology, growth, development, fecundity, and survival. Such down-stream effects of diet change also represent phenotypic plasticity [1–2].

Although, the biochemical, metabolic, and performance responses to changing diets are well documented, the gene-regulation that often underlies these downstream responses is not well-studied. In this paper, we seek to understand how diet influences gene-regulation and, subsequently, performance. Specifically, we examine the transcriptomics that underlie the manifold changes resulting from altered diets. Altered gene-regulation lies at the basis of much phenotypic plasticity [1, 3, 14]. A second goal is to understand how diet influences the biology of the rangeland grasshopper pest, *Oedaleus asiaticus* (Orthoptera: Acrididae). Our ultimate goal is to use knowledge about nutrition and diet-induced gene-regulation to control insect pests [15].

Controlling pests by manipulating their nutritional/feeding biology is reasonable [15–16]. Most phytophagous insect pests specialize on particular host plants [16–18], and this specialization determines, in part, their ecological distribution and population dynamics [19–21]. The phytophagous insect-host plant relationship is an example of co-adaptation, co-evolution, and co-speciation [22–23], with host plant suitability mainly determined by nutrition and secondary plant compounds [24–26]. For example, grasshopper species have well-defined nutritional requirements in terms of carbohydrates, lipids, proteins, vitamins, and minerals [4–5]. Consequently, they have adapted to feeding on plants with varying nutritional qualities, and can accurately choose optimal food when provided with a choice [7, 23]. The fitness and performance of herbivores increases when they feed on plants of optimal quality and fewer toxins [8–9, 25]. The availability of such plants can induce pest outbreaks, whereas blocking access to certain nutrients or otherwise disrupting digestion, assimilation, and nutritional metabolism may reduce pest-insect populations [4, 16].

Previous biological and ecological research has examined the adaptations of herbivores to their host plants. For example, diet-dependent metabolic responses of insect herbivores such as *Spodoptera spp*. have been studied by RNA-Seq analysis [10]. However, it is still unclear how host food adaptability in grasshoppers affects their physiological processes and molecular mechanisms at the gene-level. *O*. *asiaticus* specializes on grasses [27–28], and is a dominant locust in north Asian grasslands [16, 29]. Outbreaks of *O*. *asiaticus* often cause grassland damage and economic disruption [30–31]. To study phenotypic plasticity we reared *O*. *asiaticus* on four different single-plant diets, and measured resulting performance. We then sequenced and compared the transcriptomes from grasshoppers fed on each diet. Bioinformatics and differential gene expression analysis revealed that different food plants induced different gene expression profiles in *O*. *asiaticus*. These results provide new insights into the alteration of transcription by host plants and enhance our understanding of the gene expression variation underlying phenotypic plasticity in phytophagous insects.

## Materials & methods

### Ethics statement

Grasshoppers, *Oedaleus asiaticus* B. Bienko (Orthoptera: Acrididae), were field-collected at Xilin Gol grassland in 2015. Grasshoppers are common agricultural pests and are not included in the “List of Protected Animals in China”. No specific permits were required for the described field studies.

### Study sites

The research site (43.968°N, 115.821°E) was located in the Xilin Gol League, Inner Mongolia, northeastern China, a region representative of Eurasian steppe grassland [16]. The mean annual temperature in the study area is 0.3°C with mean monthly temperatures ranging from -21.6°C in January to 19.0°C in July. Air temperatures can fall as low as -41^°^C in December and reach 35^°^C in July. The mean annual precipitation is 346 mm, more than 80% of which occurs during the growing season from May to September [29, 32–33]. Vegetation at the study site is dominated by five plant species: *Cleistogenes squarrosa* (Trin.) Keng, *Leymus chinensis* (Trin.) and *Stipa krylovii* Roshev (all three are grasses: Family Poaceae), *Artemisia frigida* Willd (Asteraceae), and, *Caragana microphylla* Lam. (Leguminosea). However, in this ecosystem, plant composition is highly variable in space, and the abundance of any one plant species can change dramatically over fairly short distances. For example, *A*. *frigida* can comprise 0.3% to 29.6% of plant individuals at different sites [27, 34]. *O*. *asiaticus* grasshoppers must somehow adapt to this plant diversity. Three grasshoppers, *O*. *asiaticus* B. Bienko, *Calliptamus abbreviatus* Ikonn and *Dasyhippus barbipes* (Fischer-Waldheim) are widely distributed in this region [35]. These three species overwinter as eggs, hatch between late-May and late-June, and reach adulthood in early to late July [35].

### Feeding trials

We investigated *O*. *asiaticus* performance when reared on different host plant species during late June, 2015. A 200-m^2^ flat area of steppe was prepared by removing all vegetation using a mower. We then installed 20 gauze-covered cages (each measuring 1 m × 1 m × 1 m) in five rows with four cages in each row. The distance between cages was 1 m. We placed field-collected soil on the floor of each cage to a depth of 10 cm, and removed spiders and other natural enemies from the cages before adding *O*. *asiaticus* nymphs. The mesh covering stifled wind flow, and the cage placement gave equal exposure to sunlight, reducing microclimate differences amongst cages. Each cage was assigned to one of four treatments, consisting of one of four single plants; *C*. *squarrosa*, *L*. *chinensis*, *S*. *krylovii* or *A*. *frigida*. To start the experiment, we collected 3^rd^-instar *O*. *asiaticus* of mixed sex from the field. Thirty were immediately euthanized, dried at 90°C for 24 h, and individually weighed to establish the dry starting mass of our experimental animals. We then randomly assigned 16 individuals to each cage. Hence, each of the four treatments contained 80 individuals, divided among five replicates (cages)/per treatment, in a randomized block design.

Insects remained in their cages and received the specified feeding treatment until all individuals developed into 5^th^ instar nymphs. The experiment ran for ~30 d. Each morning, fresh plants were cut at ground level and each species was placed in a separate plastic container and returned to the laboratory. The wet weight of each plant was determined (Mettler/ML104, 0.0001 g) and 50 g of a single plant species was placed into a rectangular plastic container (20 cm × 10 cm × 2 cm) containing sterile water. The top of the container was perforated, through which the plant stems were inserted. One container was placed into each cage, and embedded in the soil so that the top of the container was flush with the soil surface. Fresh vegetation was replaced every 24 h, thus providing surplus food in a semi-natural environment. Morning feeding provided the freshest food for the grasshoppers, which fed heavily in the morning. We surveyed field cages daily to monitor survival until all surviving individuals became 5^th^ instar nymphs. They were then euthanized and weighed to obtain their dry mass using the method described above.

Development time was calculated from 3^rd^ to 5^th^ instar nymphs. Survival rate was calculated by the number surviving to 5^th^ instar/the number of initial 3^rd^ instar nymphs (n = 16) [36]. Growth rate was calculated as increase in the dry body mass/development time. Overall performance (growth rate × survival) was used to evaluate adaptability to food plants [16]. One-way analysis of variance (ANOVA) was used to compare the statistical difference between the four treatments using SAS version 8.0.

### RNA isolation and quantification

When our treatment insects reached 5^th^ instar, we analyzed their RNA. We collected 2 samples from each of our 4 treatments. Each sample consisted of 5 newly molted 5^th^ instar female nymphs (1 chosen randomly from each of the 5 replicates). Hence, in total, we analyzed 8 samples (2 from each diet). Each sample consisted of 5 female nymphs (combined) from a single diet. The collected samples were named by abbreviating the insect name and food plant followed by the sample number; OA_Sk_1, OA_Sk_2, OA_Cs_1, OA_Cs _2, OA_Lc_1, OA_Lc_2, OA_Af_1, and OA_Af_2.

Total RNA was extracted from each of the 8 samples, using the TRIzol reagent (Invitrogen, California, USA) following manufacturer instructions. The RNA sample quality was examined in 4 steps: RNA degradation and contamination was monitored on 1% agarose gels; RNA purity was checked using the NanoPhotometer spectrophotometer (IMPLEN, CA, USA); RNA concentration was measured using the Qubit RNA Assay Kit in the Qubit 2.0 Fluorometer (Life Technologies, CA, USA); and RNA integrity was assessed using the RNA Nano 6000 Assay Kit of the Agilent Bioanalyzer 2100 system (Agilent Technologies, CA, USA), respectively. The OD260/280 ratios of extracted RNA were between 1.9 and 2.1, which were deemed high quality. All samples had RNA integrity number (RIN) >8.0.

### Library preparation for transcriptome sequencing

A total of 1.5 μg RNA per sample was used as input material for the RNA sample preparations. Sequencing libraries were generated using NEBNext Ultra RNA Library Prep Kit for Illumina (NEB, USA) following manufacturer recommendations and index codes were added to attribute sequences to each sample. Briefly, mRNA was purified from total RNA using poly-T oligo-attached magnetic beads. Fragmentation was carried out using divalent cations under elevated temperature in NEBNext First Strand Synthesis Reaction Buffer (5X). First strand cDNA was synthesized using random hexamer primers and M-MuLV Reverse Transcriptase (RNase H^-^). Second strand cDNA synthesis was subsequently performed using DNA Polymerase I and RNase H. Remaining overhangs were converted into blunt ends via exonuclease/polymerase activities. After adenylation of 3’ ends of DNA fragments, NEBNext Adaptors with a hairpin loop structure were ligated to prepare for hybridization. To select cDNA fragments that were 150~200 bp in length, the library fragments were purified with AMPure XP system (Beckman Coulter, Beverly, MA, USA). Then, 3 μl USER Enzyme (NEB, USA) was used with size-selected, adaptor-ligated cDNA at 37°C for 15 min followed by 5 min at 95°C before PCR. Then, PCR was performed with Phusion High-Fidelity DNA polymerase, Universal PCR primers and Index (X) primer. Finally, PCR products were purified (AMPure XP system) and library quality was assessed on the Agilent Bioanalyzer 2100 system.

### Clustering and sequencing

Clustering of the index-coded samples was performed on a cBot Cluster Generation System using TruSeq PE Cluster Kit v3-cBot-HS (Illumia) according to the manufacturer instructions. After cluster generation, the libraries were sequenced on an Illumina HiSeq 2000 platform and 125 paired-end reads were generated.

### Quality control

Raw data (raw reads) in fastq format were first processed through an in-house perl script (Novogene Experimental Department, China). In this step, clean reads were obtained by removing adapters and reads containing ploy-N and low quality reads. At the same time, Q20, Q30, GC-content and sequence duplication level of the clean data were used for data filtering. All downstream analyses were based on clean, high quality data.

### Transcriptome assembly

The left files (read1 files) from all libraries/samples were pooled into one big left.fq file, and right files (read2 files) into one big right.fq file. Transcriptome assembly was accomplished based on left.fq and right.fq using Trinity (version: r20140413p1) with min_kmer_cov set to 2, K set to 25 by default and all other parameters set at default. Trinity [37] partitions the sequence data into many individual de Bruijn graphs, each representing the transcriptional complexity at a given gene or locus, and then processes each graph independently to extract full-length splicing isoforms and to tease apart transcripts derived from paralogous genes. Briefly, the process includes three components: Inchworm, Chrysalis, and Butterfly. Inchworm assembles the RNA-seq data into the unique sequences of transcripts, often generating full-length transcripts for a dominant isoform, but then reports just the unique portions of alternatively spliced transcripts. Chrysalis clusters the Inchworm contigs into clusters and constructs complete de Bruijn graphs for each cluster. Each cluster represents the full transcriptonal complexity for a given gene (or sets of genes that share sequences in common). Chrysalis then partitions the full read set among these disjoint graphs. Butterfly then processes the individual graphs in parallel, tracing the paths that reads and pairs of reads take within the graph, ultimately reporting full-length transcripts for alternatively spliced isoforms, and teasing apart transcripts that correspond to paralogous genes (https://github.com/trinityrnaseq/trinityrnaseq/wiki).

### Annotation of gene function

Gene function was annotated based on the following databases: Nr (NCBI non-redundant protein sequences); Nt (NCBI non-redundant nucleotide sequences); Pfam (Protein family); KOG/COG (Clusters of Orthologous Groups of proteins); Swiss-Prot (A manually annotated and reviewed protein sequence database); KO (KEGG Ortholog database) and GO (Gene Ontology).

### Quantifying gene expression levels

Gene expression levels for each sample were estimated by RSEM [38]. First, clean data were mapped back onto the assembled transcriptome, followed by obtaining the readcount for each gene from the mapping results.

### Differential gene expression analysis

Differential gene expression analysis was performed using the DESeq R package (1.10.1) to compare two feeding conditions/group [39]. DESeq provides statistical significance while determining differential expression of digital gene expression data using a model based on the negative binomial distribution. The resulting *P* values were adjusted using the Benjamini and Hochberg approach to control for false discovery rates. Genes with an adjusted *P*-value < 0.05 were assigned as differentially expressed.

### GO enrichment analysis

Gene Ontology (GO) enrichment analysis of the differentially expressed genes (DEGs) was performed using the GOseq R packages based on Wallenius non-central hyper-geometric distribution [40], which can adjust for gene length bias in DEGs.

### KEGG pathway enrichment analysis

KEGG [41] is a database resource used to understand high-level gene functions and utilities of biological systems, at the cell, organism or ecosystem level, from molecular information generated from genome sequencing and other high-throughput experimental technologies (http://www.genome.jp/kegg/). We used KOBAS [42] software to test the statistical enrichment of differentially expressed genes in KEGG pathways.

### Quantitative real-time PCR validation of RNA-Seq data

Ten candidate DEGs involved in insect cuticle biosynthesis, stress-resistant or detoxifying enzymes were chosen for validation using quantitative real-time PCR (qRT-PCR), including *CHS* (chitin synthase, c74226_g1), *CUP*2 (cuticle protein 2, c87369_g1), *LCP* (larvae cuticle protein, c84112_g1), *ESG* (endocuticle structural glycoprotein, c84444_g1), *CUP*1 (cuticular protein RR-1 motif 8, c73001_g2), *CYP* (cytochrome P450 6k1, c87438_g1), *CAT* (carboxylesterase, c82555_g1), *HSP* (heat shock protein 19.8, c88585_g1), *SBD* (sorbitol dehydrogenase, c80735_g3) and *NSO* (inositol oxygenase, c87127_g1). Gene-specific primers of those ten genes were designed using Primer Express Software v2.0 (Applied Biosystems, Foster City, CA, USA). All primers used are listed in S1 Table. Experiments were performed in the StepOne Plus Real-Time PCR system (Applied Biosystems) using SYBR green PCR mix (QIAGEN, Hilden, Germany). Then, β-actin was amplified for internal standardization. PCR efficiency and specificity of primers of the target genes were validated in the experiment. The qRT-PCR was performed in a 25 μl reaction mixture, and PCR was conducted under the following conditions: denaturation at 95 °C for 2 min, followed by 40 cycles of 94° C for 10 s, annealing at 59 °C for 10 s, and extension at 72 °C for 40 s. At the end of each reaction, the melting curve was analyzed to confirm the specificity of the primers. Relative gene expressions were normalized by the internal standard of actin, and analyzed using the 2^−ΔΔCT^ Method [43]. Expression values were adjusted by setting the expression of *O*. *asiaticus* feeding on *S*. *krylovii* to be 1 for each gene. All qRT-PCRs for each gene was performed in three technical repeats. Statistical analysis of qRT-PCR data was conducted using the ANOVA procedure of SAS 8.0.

## Results

### Phenotypic plasticity of *O*. *asiaticus* to different plant foods

**Fig 1:**
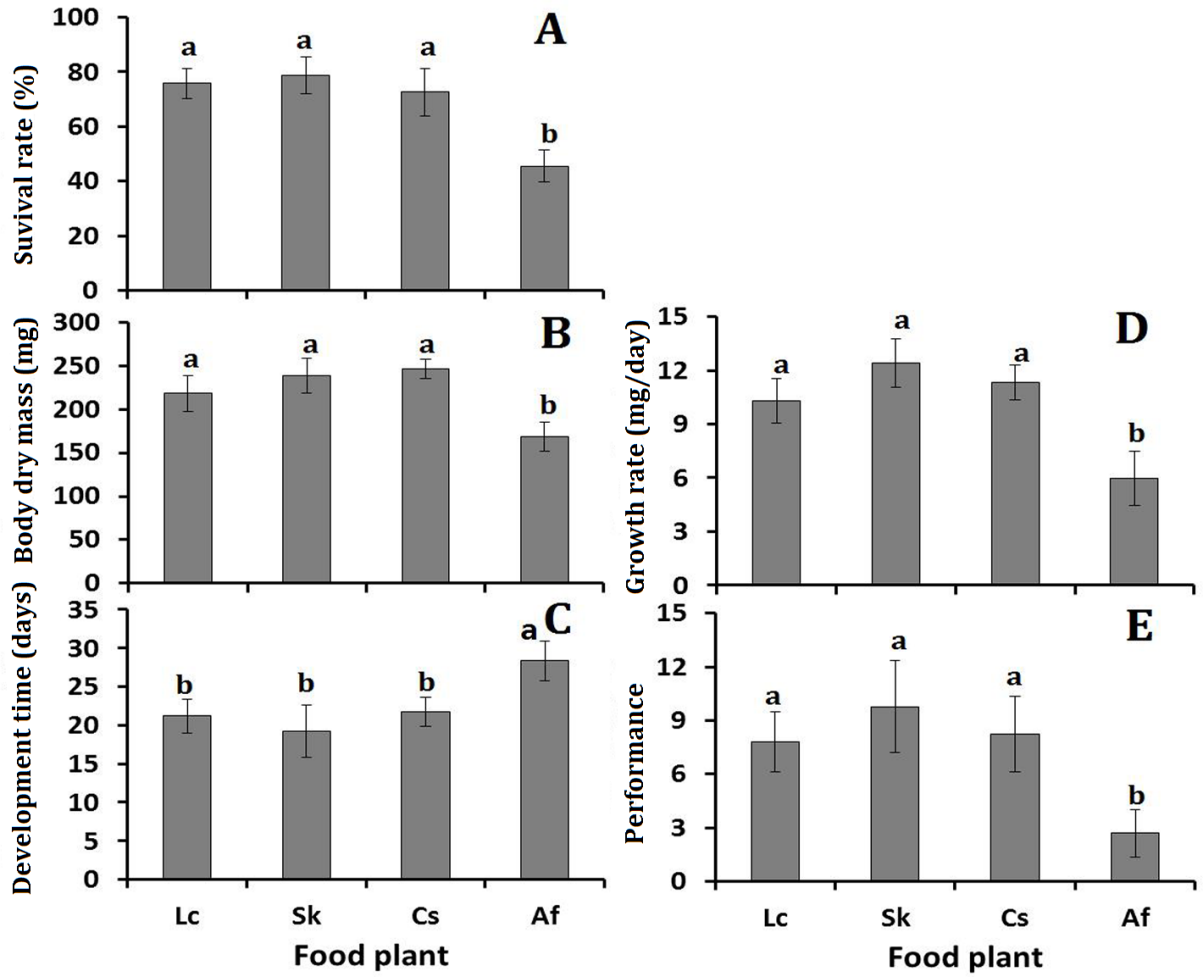
***O*. *asiaticus* mean % survival rate from 3**^**rd**^ **to 5**^**th**^ **instar ± SE (A), mean dry mass (mg ±SE) of 5**^**th**^ **instar nymphs (B), mean developmental time (days± SE) from 3**^**rd**^ **instar to 5**^**th**^ **instar (C), growth rate (mg/day ±SE) (D) and overall performance (survival rate (SR) × growth rate (GR) ±SE) (E) when fed on Lc (*L*. *chinensis*), Sk (*S*. *krylovii*), Cs (*C*. *squarrosa*) and Af (*A*. *frigida*).** Bars marked by different lowercase letters are significantly different based on Turkey’s HSD analysis at *P* <0.05.

We examined *O*. *asiaticus* phenotypic plasticity, in response to four different diets (Fig 1, S2 Table). The survival rate (Fig 1A), developmental time (Fig 1B), adult dry mass (Fig 1C), growth rate (Fig 1D), and overall performance (Fig 1E) were significantly worse for *O*. *asiaticus* feeding on *A*. *frigida*, compared to any of the three grass species (*L*. *chinensis*, *S*. *krylovi*, or *C*. *squarrosa*). This indicates that feeding on *A*. *frigida* provided less benefit for *O*. *asiaticus* growth and development, presumably because of poor adaptability to this plant compared to the other three grass species. By comparison, among the three grasses, survival rate, developmental time, adult dry mass, growth rate and overall performance, were not significantly different (Fig 1A–1E).

### Transcriptome analysis

Sequencing the transcriptomes of *O*. *asiaticus* fed on the four plant species generated approximately 73–104 million clean reads, a total of 195 million nucleotides, 223,717 transcripts, and 171,743 unigenes, with high value Q20 and Q30, a reasonable GC-content, and a low error rate from data filtering (Table 1). The N50 and N90 of transcript length were 1, 965 and 283, respectively (S1 Fig).

**Table 1:** Summary of RNA-seq metrics from *O*. *asiaticus* transcriptomes. Key: OA_Lc (*O*. *asiaticus* feeding on *L*. *chinensis*), OA_Sk (*O*. *asiaticus* feeding on *S*. *krylovii*), OA_Cs (*O*. *asiaticus* feeding on *C*. *squarrosa*) and OA_Af (*O*. *asiaticus* feeding on *A*. *frigida*), respectively.

Sample	Raw Reads	Clean reads	Clean bases	Total mapped	Error rate(%)	Q20(%)	Q30(%)	GC(%)
OA_Sk_1	88,551,484	86,812,822	13.02G	69,215,776 (79.73%)	0.02	95.64	88.96	47.71
OA_Sk_2	106,035,394	103,712,382	15.56G	81,649,610 (78.73%)	0.02	96.15	90.26	46.7
OA_Lc_1	81,423,216	79,415,554	11.91G	61,677,054 (77.66%)	0.02	95.88	89.67	47.98
OA_Lc_2	87,027,806	84,501,744	12.68G	64,345,132 (76.15%)	0.02	95.62	89.38	47.94
OA_Cs_1	74,734,806	73,066,348	10.96G	57,626,576 (78.87%)	0.02	96.01	89.96	46.05
OA_Cs_2	88,691,840	86,786,648	13.02G	69,713,986 (80.33%)	0.02	96.2	90.37	46.49
OA_Af_1	85,781,554	83,604,278	12.54G	67,285,340 (80.48%)	0.02	95.7	89.3	45.48
OA_Af_2	87,555,228	85,598,508	12.84G	69,172,726 (80.81%)	0.02	96.08	90.13	45.53
Total nucleotides	194,804,064							
Total Transcripts	223,717							
Total unigenes	171,743							
Total Annotated	45,517							

To identify the molecular mechanisms underlying these transcriptomic profiles, we compared unigene sequences to protein databases, including NCBI Nr, Swiss-Prot, KEGG, KOG and GO (e-value < 0.00001) by blastx, and to the NCBI Nt database (e-value < 0.00001). The unigenes were named and functionally annotated based on the highest sequence similarity to the retrieved proteins/genes (S3 Table). Of the 171,743 unigenes, a total of 45,517 (26.5%) were annotated in at least one database. Among them, 33,847 (19.7%) were successfully annotated by NCBI Nr, 16,759 (9.75%) by Swiss-Prot, 28,324 (16.49%) by GO, 7,571(4.4%) by KEGG, 11,700 (6.81%) by KOG, and 6,155 (3.58%) by NCBI Nt. These transcriptome data have been submitted to the SRA database in NCBI (Accession number SRP072969).

**Fig 2:**
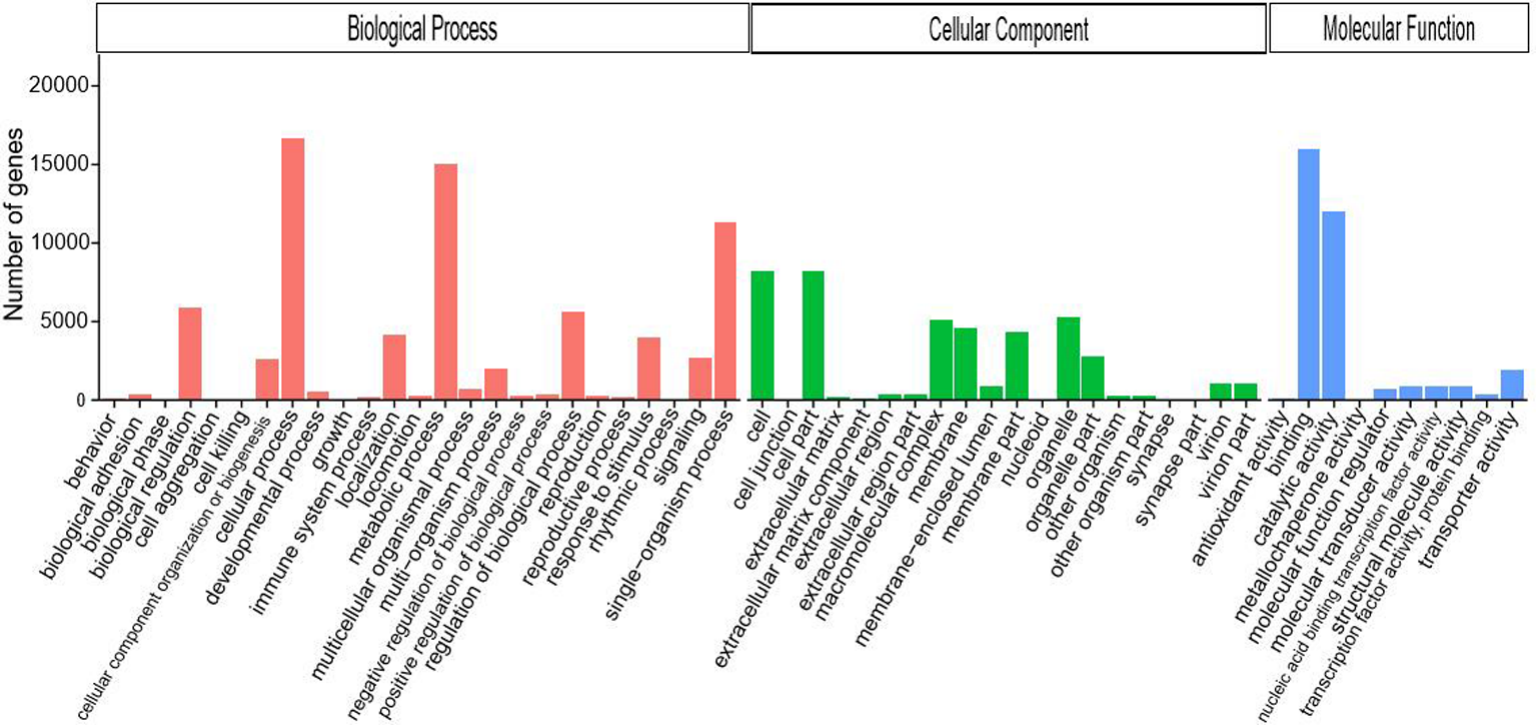
Gene Ontological classification of unigenes from *O*. *asiaticus* transcriptome. The unigenes are grouped into three hierarchically structured GO terms; biological process, cellular component and molecular function. The y-axis indicates the number of genes in each GO term.

Results of the NCBI Nr annotation (S2 Fig) showed that the majority of the sequences matched insect proteins, with the most abundant matching *Zootermopsis nevadensis* (21.4%), *Stegodyphus mimosarum* (7.8%), *Tribolium castaneum* (5.5%), *Lasius niger* (5.0%) and *Acyrthosiphon pisum* (4.5%). GO annotation (Fig 2) divided the unigenes into three functional classifications; biological process, cellular component, and molecular function. The majority of the unigenes were annotated to the following terms; cellular process, metabolic process, single-organism process, and binding and catalytic activity. KOG annotation (S3 Fig) divided the unigenes into 26 groups with the majority of unigenes annotated to the general function prediction category, followed by signal transduction mechanisms. With the KEGG annotation (S4 Fig), unigenes were divided into 267 pathways with the majority of unigenes annotated to signal transduction, carbohydrate metabolism, translation, endocrine system and transport, and catabolism.

### Differentially expressed genes (DEGs) in *O*. *asiaticus* fed on different host plants

**Fig 3:**
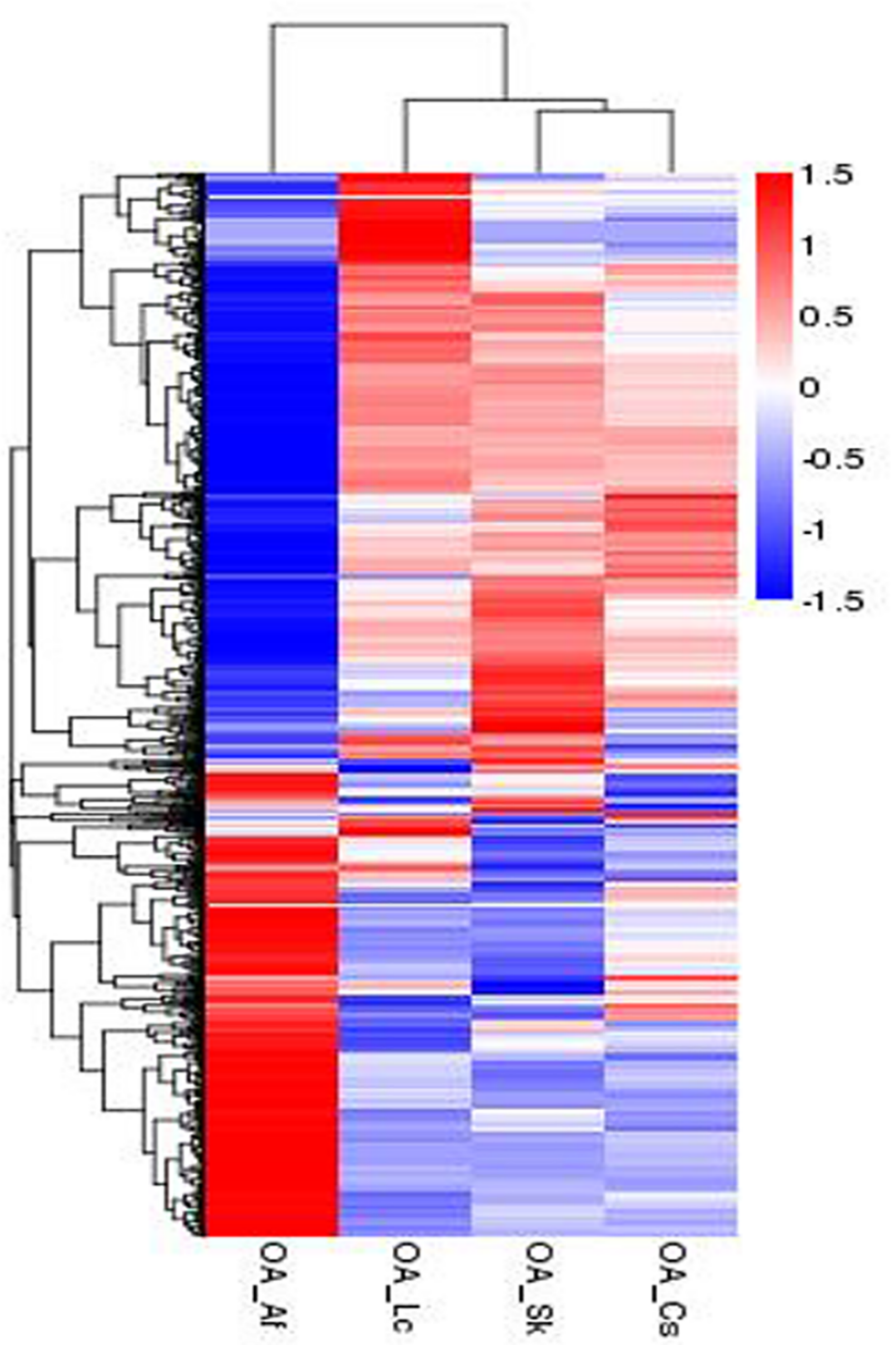
Cluster analysis of differentially expressed genes in *O*. *asiaticus* feeding on four different plant species (*A*. *frigida*, *L*. *chinensis*, *S*. *krylovii*, *C*. *squarrosa*). Blue indicates low expression and red indicates high expression. A change from red to blue indicates a decrease in value log10 (FPKM+1) from 1.5 to -1.5. Key: OA_Af (*O*. *asiaticus* feeding on *A*. *frigida*), OA_Lc (*O*. *asiaticus* feeding on *L*. *chinensis*), OA_Sk (*O*. *asiaticus* feeding on *S*. *krylovii*) and OA_Cs (*O*. *asiaticus* feeding on *C*. *squarrosa*), respectively.

**Fig 4:**
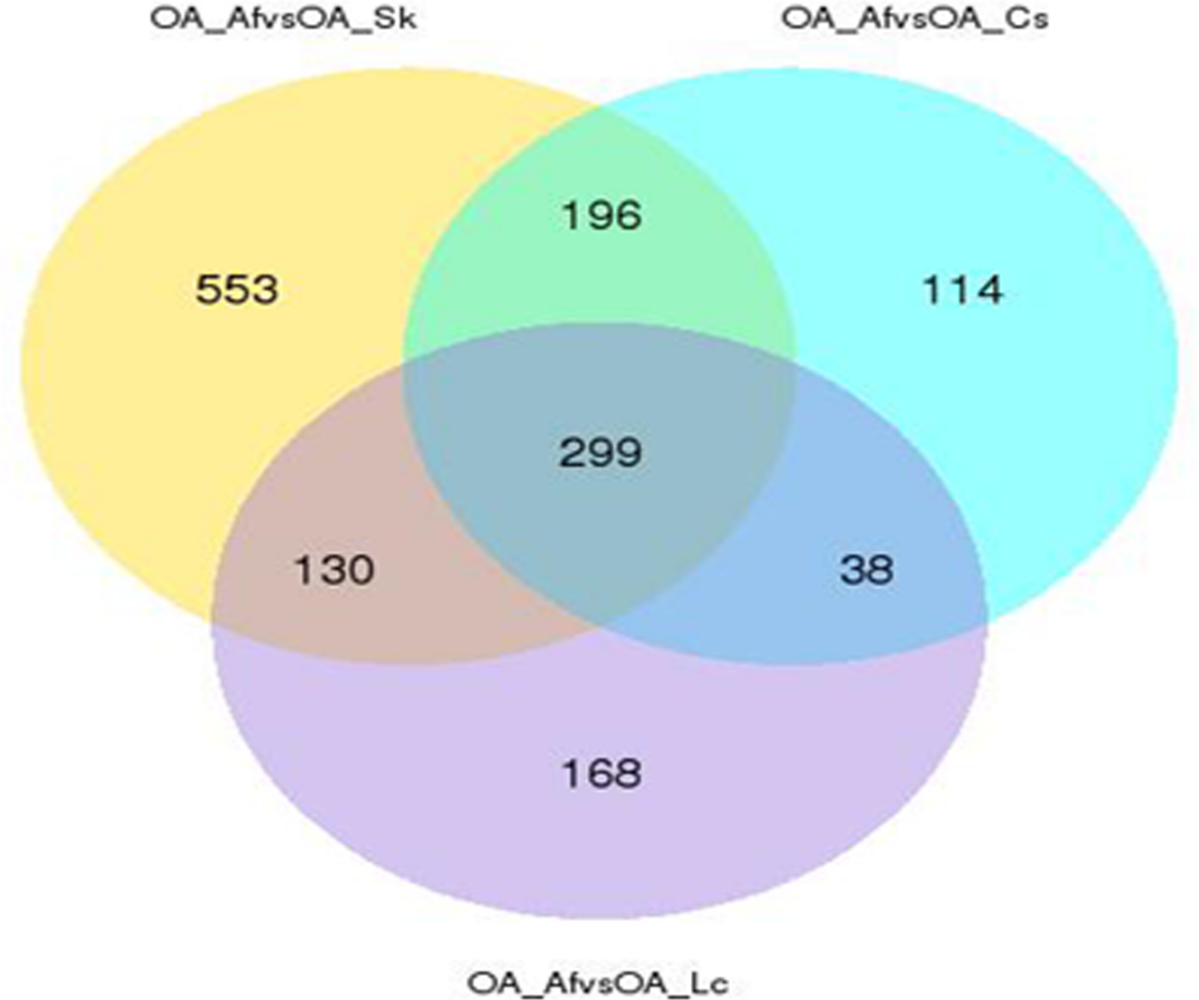
Venn diagram representing the differentially expressed genes that are similar between *O*. *asiaticus* individuals fed on the Compositae plant species, *A*. *frigida* and those that fed on graminaceous plant species, *L*. *chinensis*, *S*. *krylovii* or *C*. *squarrosa*. Key: OA_Lc (*O*. *asiaticus* feeding on *L*. *chinensis*), OA_Sk (*O*. *asiaticus* feeding on *S*. *krylovii*), OA_Cs (*O*. *asiaticus* feeding on *C*. *squarrosa*) and OA_Af (*O*. *asiaticus* feeding on *A*. *frigida*), respectively.

Our data showed that 76.15%-80.81% of the clean reads successfully mapped to the assembled transcriptome (Table 1). Differentially expressed genes (q value <0.05, |log2.Fold_change|>1) were generated by comparing OA_Af (sample feeding *A*. *frigida*) vs. OA_Sk (sample feeding *S*. *krylovii*) (690 down-regulated, 448 up-regulated), OA_Af vs. OA_Lc (sample feeding *L*. *chinensis*) (318 down-regulated, 317 up-regulated), OA_Af vs. OA_Cs (sample feeding *C*. *squarrosa*) (344 down-regulated, 303 up-regulated), OA_Lc vs. OA_Sk (50 down-regulated, 69 up-regulated), OA_Cs vs. OA_Sk (45 down-regulated, 38 up-regulated), and OA_Lc vs. OA_Cs (24 down-regulated, 71 up-regulated) (Table 2, S5 Fig). These results suggested that *O*. *asiaticus* feeding on the compositae, *A*. *frigida*, had the greatest numbers of up- or down-regulated genes compared to *O*. *asiaticus* feeding on poaceae plants, *L*. *chinensis*, *S*. *krylovii* and *C*. *squarrosa*. This was also evident from Cluster analysis of differentially expressed genes (Fig 3), which showed a marked variation between *O*. *asiaticus* fed on *A*. *frigida* compared to those that fed on the other host plants. Not surprisingly, little variation was observed in the gene expression profiles between groups that fed on different poaceae plants. We then analyzed those same differentially expressed genes (q value <0.05, |log2.Fold_change|>1) between *O*. *asiaticus* fed on *A*. *frigida* compared to those fed on the respective gramineous plants (Fig 4). The results showed that a total of 299 differentially expressed genes (196 up-regulated, 103 down-regulated) were the same among those three grass groups. The down-regulated genes mainly belonged to three functional groups (S4 Table) including insect cuticle biosynthesis (cuticular protein RR-1 motif 8, Cysteine-rich with EGF-like domain protein 2, chitin synthase 1 variant B, Cysteine-rich with EGF-like domain protein 2, *et al*.), DNA replication (such as DNA primase large subunit, endonuclease-reverse transcriptase, DNA polymerase alpha catalytic subunit, Histone H2A, DNA (cytosine-5)-methyltransferase, *et al*.), and biosynthesis and metabolism of carbohydrate (glucosyl glucuronosyl transferase, 6-phosphogluconate dehydrogenase, glucose dehydrogenase, alcohol dehydrogenase, oligosaccharyltransferase complex subunit ostc-B, *et al*.), fat (such as lipoyltransferase 1, Putative fatty acyl-CoA reductase, carnitine O-palmitoyltransferase 1, myelin expression factor 2, *et al*.) and proteins (such as Golgi integral membrane protein 4, peptidyl-prolyl isomerase-1, protein disulfide-isomerase, E3 ubiquitin-protein ligase UHRF1-like, venom dipeptidyl peptidase 4 isoform X2, *et al*.). This suggested that feeding on *A*. *frigida* could result in decreased biosynthesis activity and metabolism in *O*. *asiaticus*. Among the up-regulated genes (S5 Table), some were stress-resistant or detoxifying enzymes, such as heat shock protein 19.8, cytochrome P450 6k1, carboxylesterase, sorbitol dehydrogenase and apoptosis inhibitor IAP. This suggested that feeding on *A*. *frigida* could activate many stress-resistance and detoxification related genes.

**Table 2:** *O*. *asiaticus* differentially expressed genes (DEGs) following feeding on the four host plant species with q value <0.05.

Host plant comparison	Down-regulated genes	Up-regulated genes
*A*. *frigida* vs *S*. *krylovii*	690	448
*A*. *frigida* vs *C*. *squarrosa*	344	303
*A*. *frigida* vs *L*. *chinensis*	318	317
*L*. *chinensis* vs *S*. *krylovii*	50	69
*L*. *chinensis* vs *C*. *squarrosa*	24	71
*C*. *squarrosa* vs *S*. *krylovii*	45	38

### GO and KEGG pathway enrichment

From DEGs analysis, we found that the gene expression profiles varied significantly between *O*. *asiaticus* that fed on *A*. *frigida* and those that fed on poaceae plants. There was very little variation between nymphs that fed on poaceae plants. In the GO (Corrected *P*-value < 0.05) and KEGG enrichment (qValue < 0.05) analyses we compared DEGs from *O*. *asiaticus* that had fed *A*. *frigida* and the three poaceae plants (Table 3 and Table 4). With GOseq R packages, the majority of differentially expressed genes between *O*. *asiaticus* that fed on *A*. *frigida* and *O*. *asiaticus* fed on the three poaceae species were assigned to 23 GO terms belonging to three broad GO categories i.e. biological process (BP), cellular component (CC) and molecular function (MF) (Table 3). Down-regulated GO terms included structural constituent of cuticle (MF), chitin binding (MF), structural molecule activity (MF), fatty acid biosynthetic process (BP), carbohydrate metabolic process (BP), small molecule catabolic process (BP) and oligosaccharyltransferase complex (CC). This suggested that *O*. *asiaticus* capacity for biosynthesis and metabolism significantly decreased when fed on *A*. *frigida*. Up-regulated GO terms included regulation of signal transduction (BP), regulation of cell communication (BP), regulation of signaling (BP), inositol catabolic process (BP), alcohol catabolic process (BP), polyol catabolic process (BP) and organic hydroxy compound catabolic process (BP). This suggests that *O*. *asiaticus* fed *A*. *frigida* had increased signal transduction and metabolized other substances, such as hydroxyl compounds.

**Table 3:** GO enrichment analysis (Corrected P-value < 0.05) of the differentially expressed genes of *O*. *asiaticus* fed on *A*. *frigida* compared to those fed on *L*. *chinensis*, *S*. *krylovii* or *C*. *squarrosa*. Key: OA_Af (*O*. *asiaticus* feeding on *A*. *frigida*), OA_Cs (*O*. *asiaticus* feeding on *C*. *squarrosa*), OA_Lc (*O*. *asiaticus* feeding on *L*. *chinensis*) and OA_Sk (*O*. *asiaticus* feeding on *S*. *krylovii*), respectively. ‘-’ indicates the corrected P-value > 0.05 and therefore not significantly different.

Ontology	Class	Up-/Down- regulation	Gene number for OA_Af vs OA_Cs	Gene number for OA_Af vs OA_Lc	Gene number for OA_Af vs OA_Sk
Biological process	fatty acid biosynthetic process	Down	8	7	13
	carbohydrate metabolic process	Down	19	31	47
	small molecule catabolic process	Down	8	6	11
	organic acid catabolic process	Down	7	-	10
	steroid metabolic process	Down	6	6	-
	carboxylic acid catabolic process	Down	-	11	9
	glycerol-3-phosphate metabolic process	Down	10	7	12
	valine metabolic process	Down	6	-	7
	regulation of signal transduction	Up	9	6	9
	regulation of cell communication	Up	8	-	6
	regulation of signaling	Up	9	7	-
	inositol catabolic process	Up	4	4	3
	alcohol catabolic process	Up	-	4	4
	polyol catabolic process	Up	4	5	4
	organic hydroxyl compound catabolic process	Up	-	4	4
Molecular function	structural molecule activity	Down	28	24	52
	chitin binding	Down	7	6	9
	structural constituent of cuticle	Down	14	14	28
	coenzyme binding	Down	13	-	22
	phosphogluconate dehydrogenase (decarboxylating) activity	Down	4	-	5
	transferase activity, transferring acyl groups	Down	-	-	17
Cellular component	endoplasmic reticulum	Down	7	17	12
	oligosaccharyltransferase complex	Down	5	4	-

**Table 4:** KEGG enrichment analysis (qValue < 0.05) of the differentially expressed genes in *O*. *asiaticus* fed on *L*. *chinensis*, *S*. *krylovii*, *C*. *squarrosa* or *A*. *frigida*. Key: OA_Af (*O*. *asiaticus* feeding on *A*. *frigida*), OA_Cs (*O*. *asiaticus* feeding on *C*. *squarrosa*), OA_Lc (*O*. *asiaticus* feeding on *L*. *chinensis*) and OA_Sk (*O*. *asiaticus* feeding on *S*. *krylovii*), respectively. ‘-’ indicates a corrected P-value > 0.05 and therefore not significantly different.

Pathway	Up-/Down-regulation	Gene number for OA_Af vs OA_Cs	Gene number for OA_Af vs OA_Lc	Gene number for OA_Af vs OA_Sk
DNA replication	Down	9	8	10
Fatty acid degradation	Down	10	7	9
N-Glycan biosynthesis	Down	6	5	9
Protein processing in endoplasmic reticulum	Down	12	9	17
Fatty acid metabolism	Down	15	12	16
Carbon metabolism	Down	14	-	13
Meiosis	Down	6	5	6
Protein digestion and absorption	Down	9	9	12
Cutin, suberine and wax biosynthesis	Down	8	-	9
Cell cycle	Down	5	6	-
Various types of N-glycan biosynthesis	Down	-	6	7
HIF-1 signaling pathway	Up	6	5	7
Metabolism of xenobiotics by cytochrome P450	Up	10	8	11
Rap1 signaling pathway	Up	6	5	-
FoxO signaling pathway	Up	-	4	6
Inositol phosphate metabolism	Up	-	6	6

With KOBAS software, the majority of differentially expressed genes between the *O*. *asiaticus* fed *A*. *frigida* and those fed on the three grasses were assigned to 16 (qValue< 0.05) pathways (Table 4). The down-regulated pathways mainly included DNA replication, protein processing in endoplasmic reticulum, N-Glycan biosynthesis, fatty acid degradation, cutin, suberine and wax biosynthesis, fatty acid metabolism, carbon metabolism, etc. suggesting that the ability of *O*. *asiaticus* for biosynthesis and metabolism significantly decreased after feeding on *A*. *frigida*. The up-regulated pathways mainly included the HIF-1 signaling pathway, FoxO signaling pathway, inositol phosphate metabolism, Rap1 signaling pathway, metabolism of xenobiotics by cytochrome P450, and insulin signaling pathway. These suggest that *O*. *asiaticus* fed *A*. *frigida* had increased activities in signal transduction, stress-resistance and detoxification enzymes similar to the GO analysis.

### Verification of the gene expression through qRT-PCR

**Fig 5:**
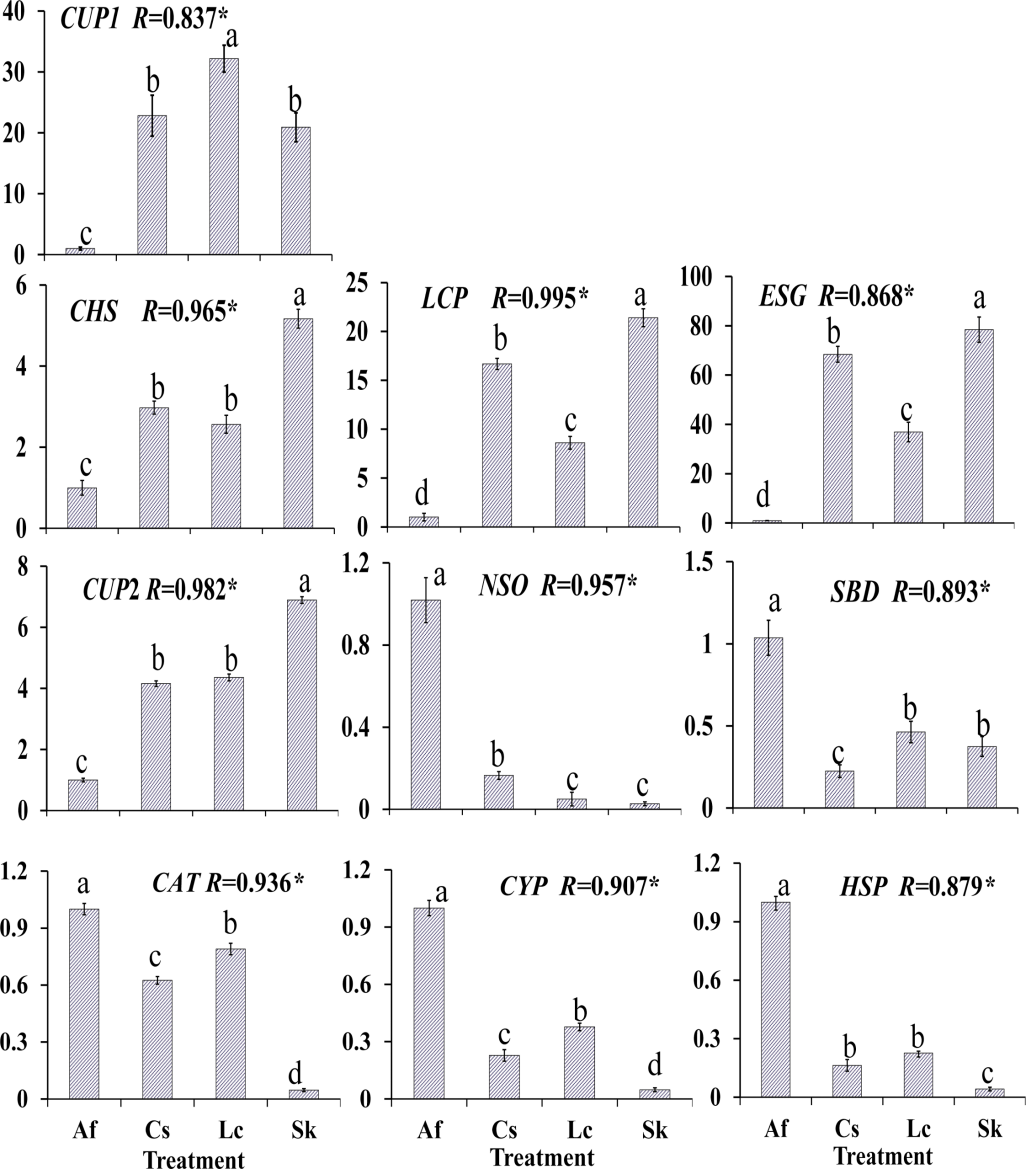
Real-time quantitative qRT-PCR confirmation of ten candidate genes. The left y-axis indicates relative gene expression levels (±SE) determined by qRT-PCR when *O*. *asiaticus* fed on Lc (*L*. *chinensis*), Sk (*S*. *krylovii*), Cs (*C*. *squarrosa*) and Af (*A*. *frigida*). Bars marked by different lowercase letters are significantly different based on Turkey’s HSD analysis at *P* <0.05. The correlation coefficient (*R*) for each gene between the RT-qPCR and RNA-Seq data is shown with the significant level (*P <0.05).

The qRT-PCR results for all ten candidate genes were tested statistically, and the cuticle biosynthesis related *CHS*, *CUP*2, *LCP*, *ESG* and *CUP*1 were significantly down-regulated in *O*. *asiaticus* that fed on *A*. *frigida* (*P* < 0.05, Fig 5). On the contrary, the stress-resistant or detoxifying enzyme related *CYP*, *CAT*, *HSP*, *SBD* and *NSO* were significantly up-regulated (*P* < 0.05). Moreover, ten genes showed significant correlations (*P* < 0.05) between the RT-qPCR data and the RNA-seq results, which indicated good reproducibility between transcript abundance assayed by RNA-seq and the expression profile revealed by qRT-PCR data.

## Discussion

In our experiment, we employed single-plant feeding trials to compare the suitability of four different food plants for *Oedaleus asiaticus* grasshoppers. Our results demonstrate that the *Artemisia frigida* (family Asteraceae), is less suitable for *O*. *asiaticus*, in comparison to three grasses (*Leymus chinensis*, *Stipa karylovii*, and *Cleistogenes squarrosa*). Grasshoppers fed only on *A*. *frigida* had reduced size, growth, development, and survival, in comparison to those fed on any of the three grass species. These results confirm previous studies [36, 44–45]. In addition, previous research showed that consumption and preference of these four plants for individuals developing from 4^th^ instar through to maturity was lowest for *A*. *frigida* [46]. Subsequent transcriptomic analysis demonstrated that insects from the three grass-fed treatments had fairly similar gene expression profiles. In contrast, *A*. *frigida*-fed grasshoppers exhibited dramatically different transcription profiles from grass-fed insects. What does this mean? Why would feeding on an unsuitable plant dramatically alter transcription profiles, and what are the consequences of such changes? These questions are best addressed under the theory of phenotypic plasticity.

Phenotypic plasticity occurs when an individual changes its phenotype. All living things can undergo phenotypic plasticity, which can be expressed as changes to biochemistry, metabolism, physiology, morphology, development, behavior, or life-history, etc. [1–2]. Altered transcription represents phenotypic plasticity because it alters the phenotype. Indeed, transcription may underlie most phenotypic plasticity [1, 14]. Small transcriptional adjustments can produce dramatic down-stream changes to phenotypes.

A confusing aspect of transcriptomic studies is that altered transcription in response to changed environments can range from highly evolved and beneficial responses, to non-evolved responses, whose outcome might be beneficial, neutral, or highly detrimental to the organism [1, 47]. Sorting out those aspects of altered transcription is difficult, because of the pleiotropic and interactive effects of any single transcription event—a single enzyme may influence numerous other enzyme pathways, substrates and products, and subsequently alter numerous divergent physiological, developmental, and morphological aspects. Some of these manifold and interacting changes may be beneficial and others detrimental to the organism [1–3]. Even seemingly harmful consequences that result from altered transcription may in fact be beneficial. An example is a transcriptional change that delays growth, development, or reproduction. This response may at first appear to be detrimental to the organism, but, in fact, may be beneficial if it allows the individual to survive during a period of stress, such as during poisoning or poor nutrition. Hence, at this time, we cannot know the ultimate fitness value or the selection history of most altered gene expression.

However, we can still draw some broad conclusions from our study. First is that different food plants induce different gene expression profiles. This confirms previous studies linking changed transcription to changed environments [10]. Second is that stress substantially altered gene expression; i.e., grasshoppers fed *A*. *frigida* exhibited at least 1, 138 differently expressed genes in comparison to grass-fed insects. This agrees with previous studies showing increased transcription with increased stress [48]. In our case, we know that feeding on *A*. *frigida* was stressful, because it significantly lowered performance. Third, we suggest that the 196 unique up- and 103 down-regulated genes in the *A*. *frigida*-fed grasshoppers produced a different animal than the grass-fed grasshoppers (i.e., feeding on *A*. *frigida* produced a population with a different phenotype). This idea is supported by the significant morphological /performance differences between grass- and *A*. *frigida*-fed grasshoppers; the two groups have significantly different phenotypes. Considering that untold environmental and social factors can alter transcription [14], this suggests that we should not view organisms as individuals whose phenotypes are fixed, but, instead, as individuals whose phenotypes are always rapidly changing in space and time. This idea has substantial theoretical consequences [14, 49].

In regards to specific genes: feeding on *A*. *frigida* resulted in down-regulation of some genes related to insect cuticle biosynthesis, DNA replication, and biosynthesis and metabolism of carbohydrate, fat and protein, and up-regulation of some genes related to stress-resistance or detoxification enzymes such as heat shock protein (Hsp), cytochrome P450, and carboxylesterase and apoptosis inhibitor. Likewise, GO and KEGG enrichment of *A*. *frigida*-fed insects indicated altered transcription of biological processes and biosynthesis and metabolism pathways, including down-regulation of structural constituents to cuticle production and carbohydrate metabolic process, and up-regulation of many biological processes and pathways related to stress-resistance and detoxification enzymes (e.g. metabolism of xenobiotics by cytochrome P450, FoxO signaling pathway, inositol phosphate metabolism, and inositol catabolic process). The altered transcriptome offers insights into the observed biological performance of *O*. *asiaticus*. Many of these plastic changes are probably responses to stress. The fact that *O*. *asiaticus* grasshoppers up-regulated stress-resistance genes after feeding on *A*. *frigida* is not surprising, given that this plant is less preferred [36, 44–45], that the *A*. *frigida*-fed insects were stressed, as indicated by significantly lower performance, and that *A*. *frigida* contains potent toxins, including terpenoids and sesquiterpene lactones [50]. As previously mentioned, animals often up-regulate detoxifying enzymes, such as cytochrome P450, in response to poisoning [51]. Heat-shock proteins are also well known to be highly inducible, and to ameliorate stress [52]. Up-regulation of both of these genes is clearly beneficial for the grasshoppers, and probably represents evolved (adaptive) phenotypic plasticity. In contrast, at this time, we do not know if the down-regulation of cuticle biosynthesis, DNA replication, and metabolism of carbohydrate, fat and protein metabolism is beneficial, detrimental, adaptive, or simply a susceptibility or accidental by-product of diet or stress.

Studies on the co-adaptation, co-evolution and co-speciation between herbivores and their host plants have provided an understanding of the behavioral, physiological, chemical, genetic, ecological and evolutionary mechanisms involved in these interactions [22, 28]. Herbivores have specific adaptability to different host plants based on a number of factors including habitat, food location, and identification, larval feeding, detoxification, growth, defense against predaceous, parasitic, or competitive enemies, and mate-finding and reproduction. Some plant species are strongly attractive to specific herbivores thus contributing to, or even accelerating, pest population outbreaks [22–23, 53]. An example is *Locusta migratoria manilensis* (Meyen), a species where outbreaks correlate with the distribution of the host plant, *Phragmites australis* (Cav.) [54–55]. Many studies have examined herbivore food adaptation based on biological and ecological preferences [23]. In this study, we contrasted altered transcription profiles after feeding on suitable vs. unsuitable plants, in order to understand the plastic genetic response of insects to variable diets.

The question of which selective factors have driven the evolution of host adaptability by insect herbivores is an area of research interest. Although ecological factors such as susceptibility to predation and other aspects of habitat association have been identified as important in selection, plant chemistry is critical, including nutrition, nutritional barriers, and secondary compounds [26,28, 56–58]. Phenotypic plasticity of individuals, genetic variability of populations, and detoxification mechanisms are just some of the mechanisms allowing herbivorous insects to overcome plant defenses and variable diets [1, 51, 59].

Specifically, it is a widely accepted hypothesis that the evolution of diet choice and food adaptability is tightly correlated with nutrition metabolism-related enzymes [60]. For example, the expression of the related proteases, lipases, mannosidases, glucosidases, and alpha amylases, enzymes responsible for starch breakdown, are differentially induced for different plants [10, 61]. For example, better survival and fecundity of the caterpillar *Hyposidra infixaria* (Lepidoptera: Geometridae), when reared on artificial diet compared to tea leaves, was linked to higher activity of nutrition metabolism-related enzymes [6]. This was also supported by our transcriptomics analysis, where 34 nutrition metabolism-related genes were up-regulated in individuals feeding on the three Poaceae (preferred host), compared to those feeding on *A*. *frigida* less preferred host).

In addition, the evolution of diet choice and food adaptability is tightly correlated with detoxication related enzymes [10]. The expression of cytochrome P450, glutathione transferase, carboxylesterase, *et al*. are differentially induced for various plants in herbivorous insect [10, 51]. When confronted with host plants having low suitability, the expression of detoxification-related enzymes, such as cytochrome P450 and glutathione transferase and carboxylesterase may be activated in response to the presence of toxic substances in the plant [51]. This was also supported by our transcriptomics analysis, where we found that the gene expression of carboxylesterase and Cytochrome P450 6k1 were up-regulated when *O*. *asiaticus* fed on the less preferred plant *A*. *frigida*.

In our experiment, we analyzed 2 samples from each treatment for RNA. Although each sample contained 5 pooled insects (hence 10 insects total analyzed/treatment), analyzing more samples, or individuals (instead of groups), would have allowed a statistical analysis of transcriptomic variance among treatments [62–64]. Today, declining costs for RNA analysis allows analysis of multiple samples.

Lastly, we would like to comment on the role of plant primary and secondary metabolites in mediating plant–insect interactions [59]. Future studies should address which plant chemicals regulate grasshopper feeding. For example, grasses may contain important nutrients, secondary metabolites, or other substances [34, 65–66], which may be critical for the development or reproduction of *O*. *asiaticus*. In addition, we need to examine exactly how the terpenoids and sesquiterpene lactones present in *A*. *frigida* influence physiology and biology. Analysis of quantitative relations between chemical plant traits (nutrition and secondary compounds) and metabolism/detoxication related gene-expression provide the opportunity for ongoing molecular research to decipher the biological mechanisms of herbivorous insect host choice and adaptability.

## Supporting information

S1 Fig Transcript length distribution from transcriptome assembly. (TIF)

S2 Fig Species classification of Nr annotation for *O*. *asiaticus* sequences. (TIF)

S3 Fig EuKaryotic Ortholog Groups (KOG) of unigenes for *O*. *asiaticus* sequences. The unigenes are grouped into 26 hierarchically structured KOG terms. The y-axis indicates the number of genes in each KOG.
(TIF)

S4 Fig Kyoto Encyclopedia of Genes and Genomes (KEGG) of unigenes for *O*. *asiaticus* sequences. KEGG analysis divided pathways into five groups, A-Cellular Processes, B-Environmental Information Processing, C-Genetic Information Processing, D-Metabolism, E-Organismal Systems. The x-axis indicates the number and percent of genes in each KEGG pathways.
(TIF)

S5 Fig Different expreseed genes analysis between *O*. *asiaticus* feeding four different plant species. Genes were divided among three classes: red genes are up-regulated in the right sample vs. the left sample, green genes are down-regulated in the right sample vs. the left sample, and blue genes are not differentially expressed. OA_Lc, OA_Sk, OA_Cs, OA_Af were representive of *O*. *asiaticus* individuals feeding *L*. *chinensis*, *S*. *krylovii*, *C*. *squarrosa*, *A*. *frigida*, respectively.
(TIF)

S1 Table Designed sequences of qRT-PCR primers for ten candidate genes. (DOCX)

S2 Table *O*. *asiaticus* survival rate from 3^rd^ to 5^th^ instar ± SE, mean dry mass (mg ±SE) of 5^th^ instar nymphs, mean developmental time (days± SE) from 3^rd^ instar to 5^th^ instar, growth rate (mg/day ±SE) and overall performance (survival rate (SR) × growth rate (GR) ±SE) when fed on Lc (*L*. *chinensis*), Sk (*S*. *krylovii*), Cs (*C*. *squarrosa*) and Af (*A*. *frigida*). (DOCX)

S3 Table Annotation results of unigenes. (DOCX)

S4 Table The same differentially expressed genes (qvalue <0.05, |log2.Fold_change|>1, only annotated and down-regulated genes) of *O*. *asiaticus* feeding *A*. *frigida* compared with individuals feeding the other three plants *L*. *chinensis*, *S*. *krylovii*, *C*. *squarrosa*. (DOCX)

S5 Table The same differentially expressed genes (qvalue <0.05, |log2.Fold_change|>1, only annotated and up-regulated genes) of *O*. *asiaticus* feeding *A*. *frigida* compared with individuals feeding the other three plants *L*. *chinensis*, *S*. *krylovii*, *C*. *squarrosa*. (DOCX)
