# Supplementary figures and images for: Diet alters performance and transcription patterns in Oedaleus asiaticus (Orthoptera: Acrididae) grasshoppers
Source: PLoS One. 2017 Oct 12;12(10):e0186397. doi: 10.1371/journal.pone.0186397 (PMC5638516; doi:10.1371/journal.pone.0186397)

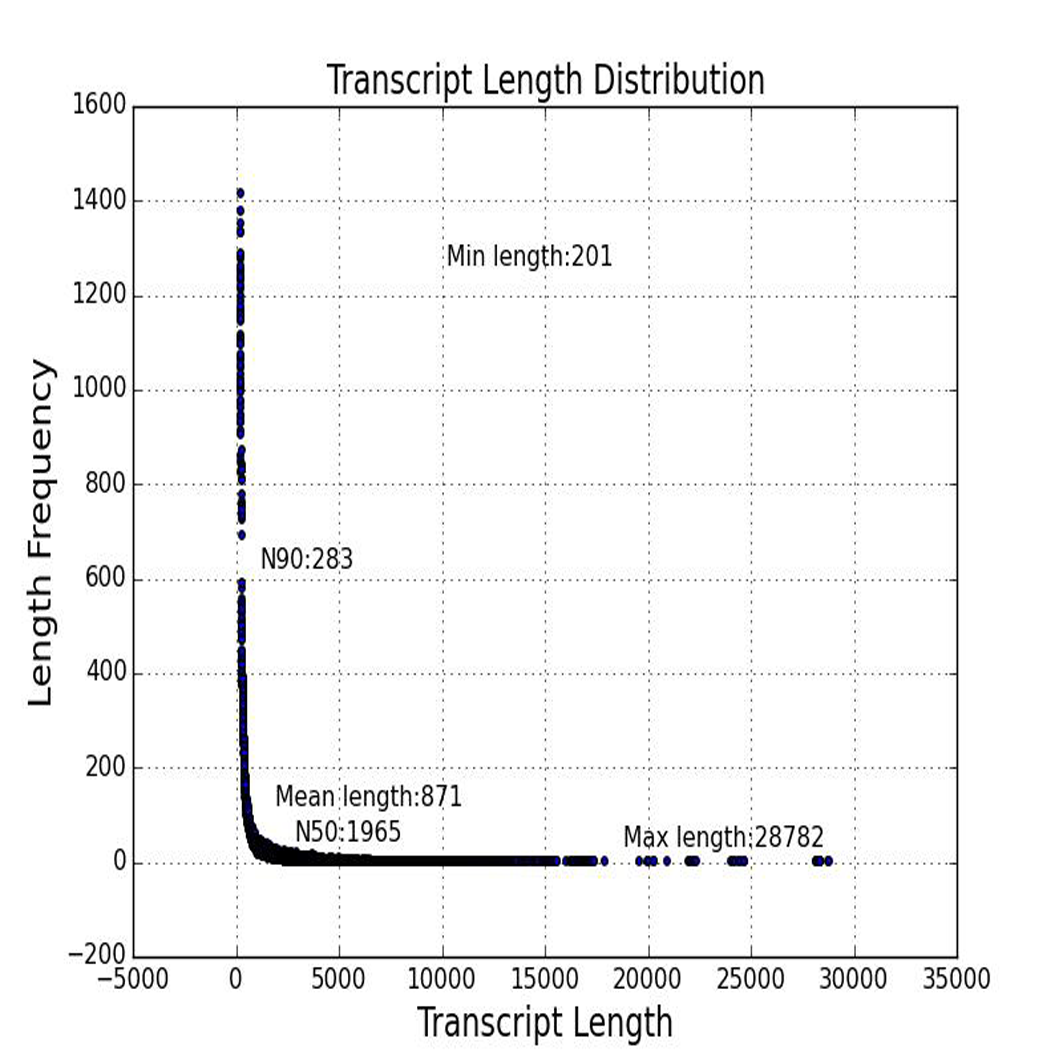

Supplement: S1 Fig — (TIF) [file pone.0186397.s001.tif]

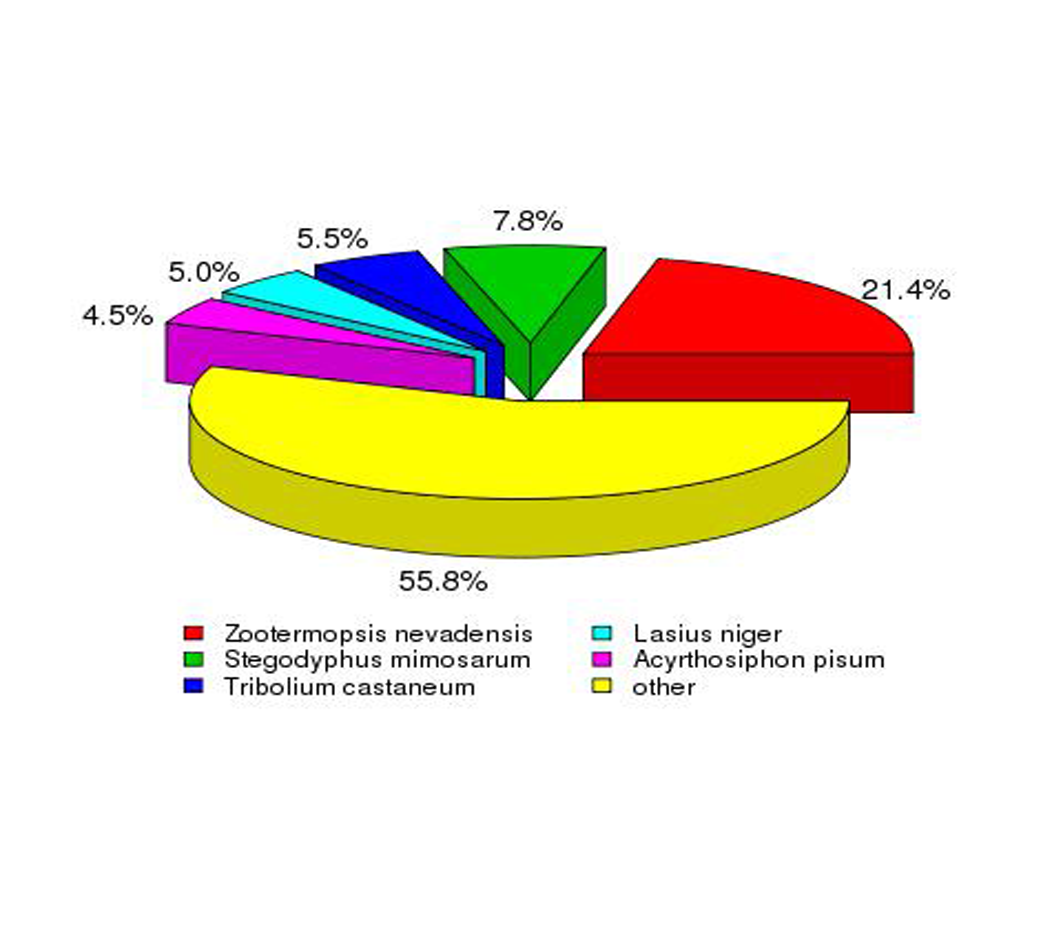

Supplement: S2 Fig — (TIF) [file pone.0186397.s002.tif]

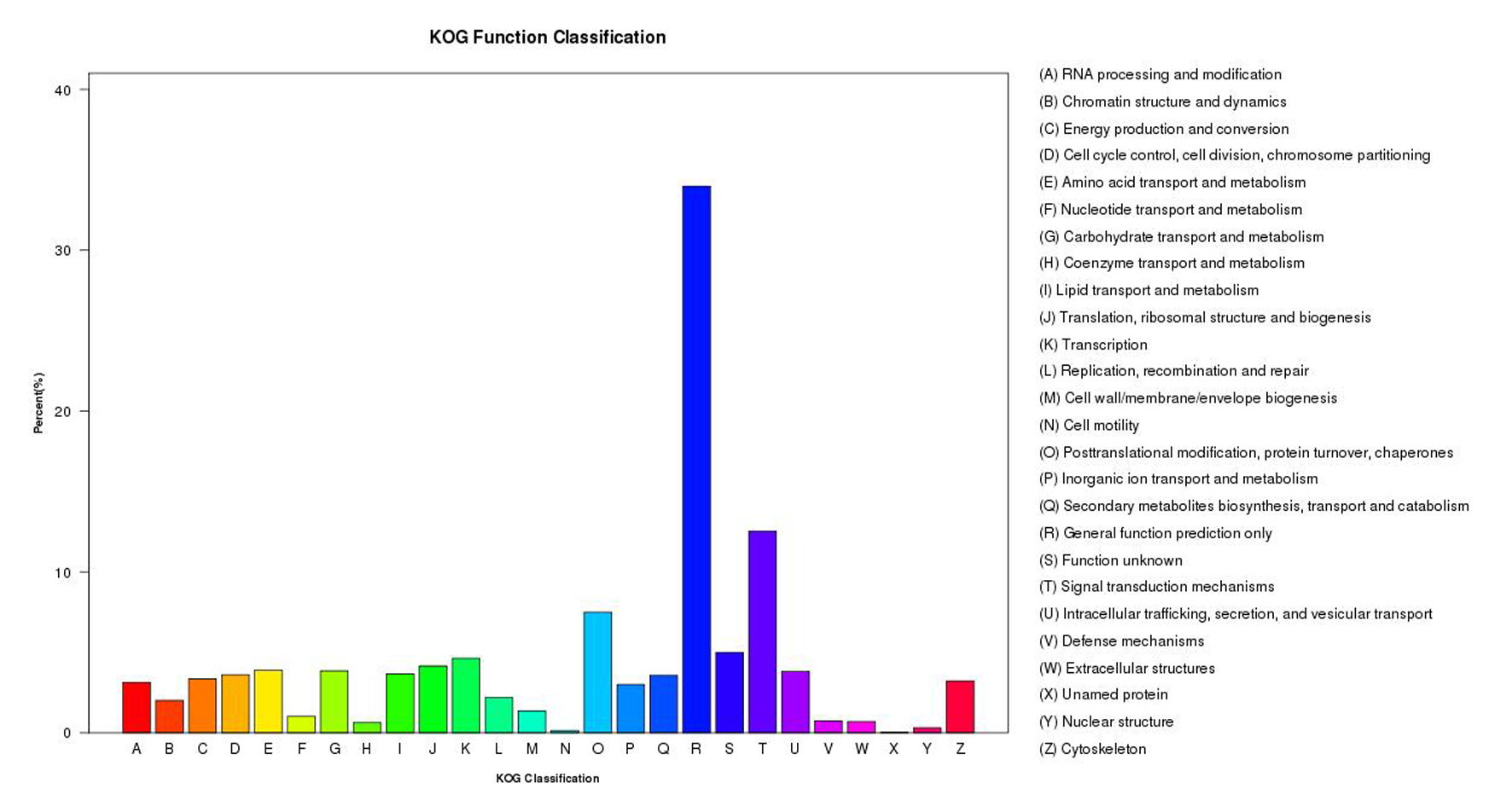

Supplement: S3 Fig — The unigenes are grouped into 26 hierarchically structured KOG terms. The y-axis indicates the number of genes in each KOG. (TIF) [file pone.0186397.s003.tif]

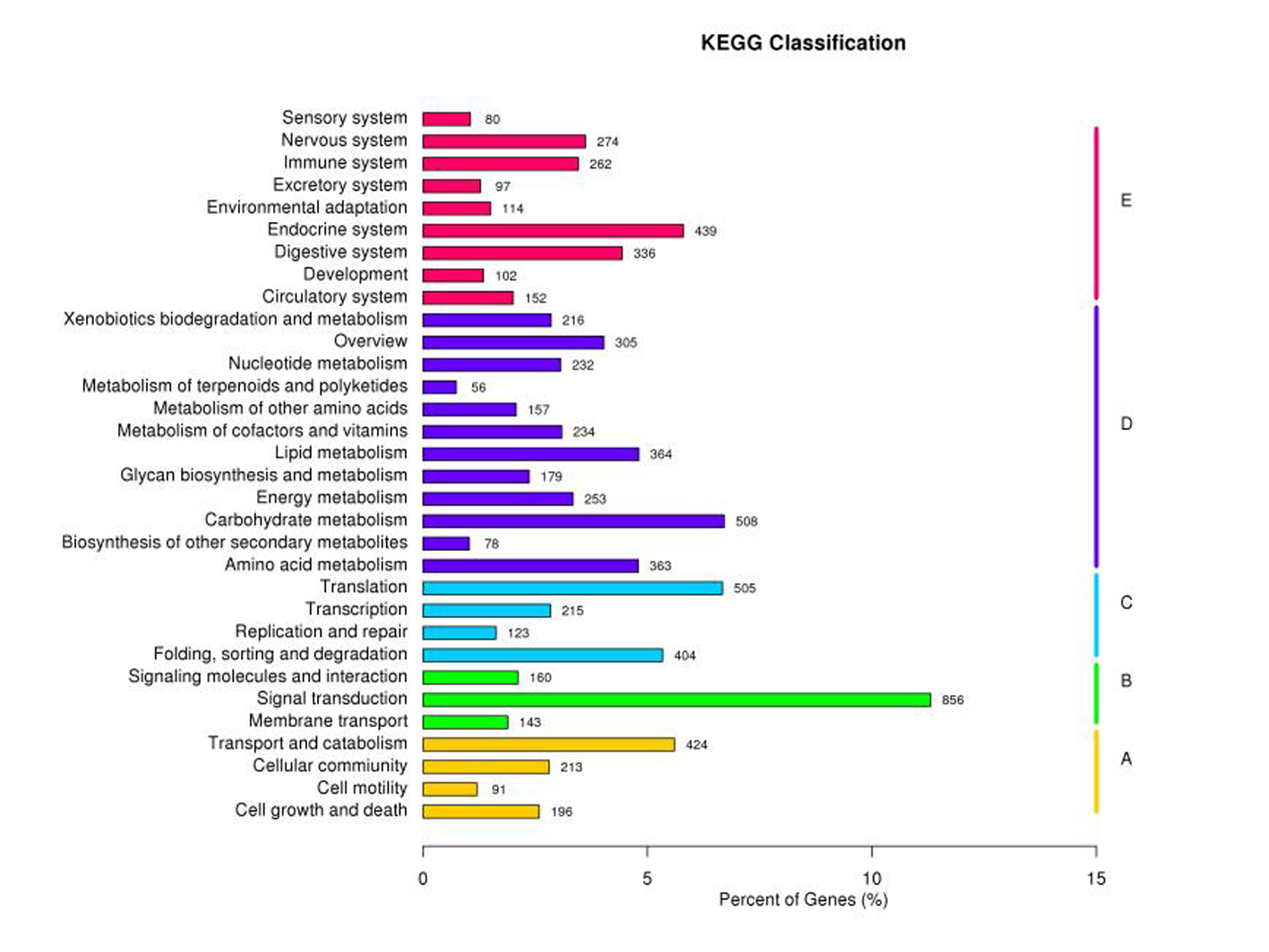

Supplement: S4 Fig — KEGG analysis divided pathways into five groups, A-Cellular Processes, B-Environmental Information Processing, C-Genetic Information Processing, D-Metabolism, E-Organismal Systems. The x-axis indicates the number and percent of genes in each KEGG pathways. (TIF) [file pone.0186397.s004.tif]

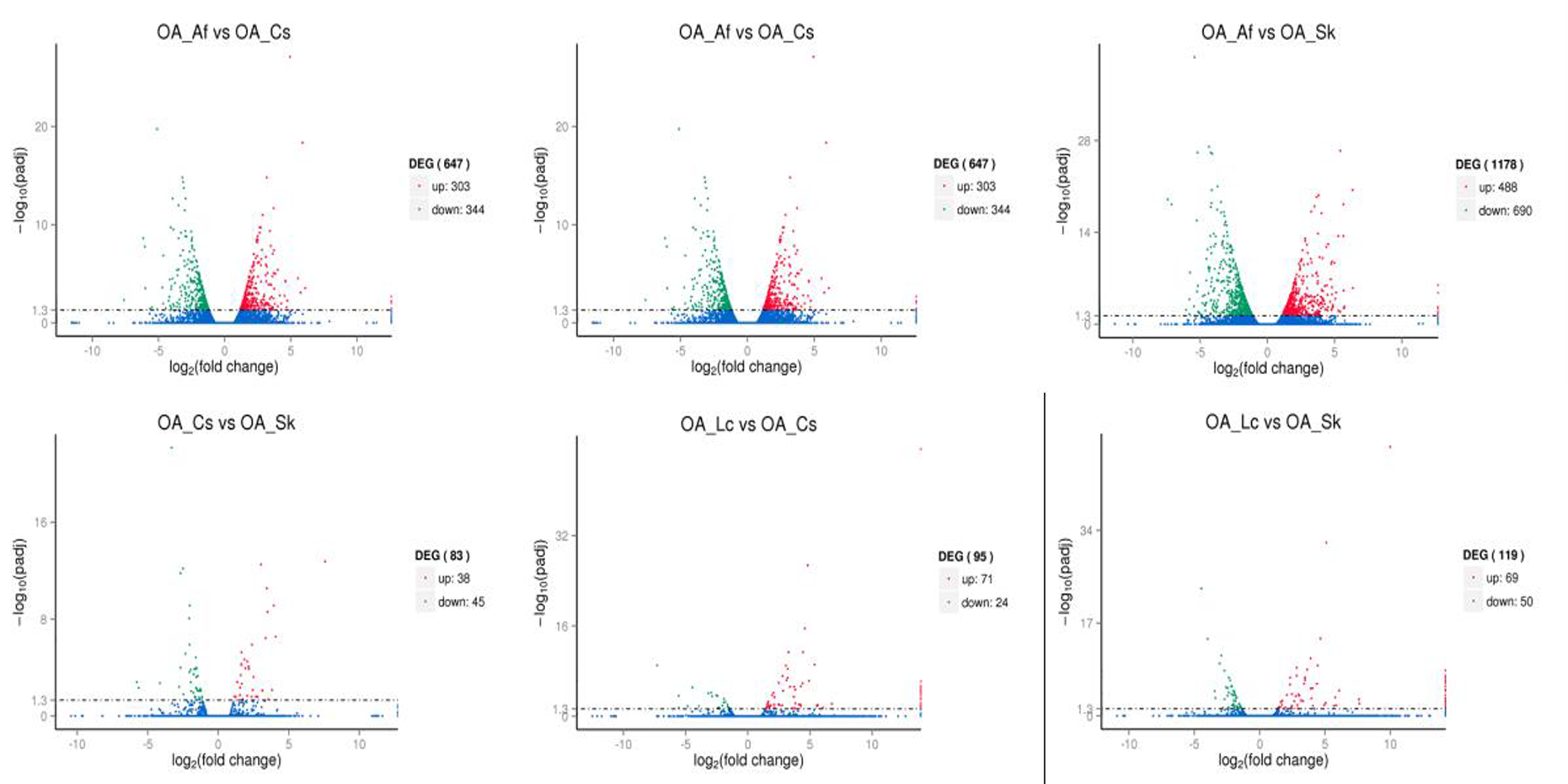

Supplement: S5 Fig — Genes were divided among three classes: red genes are up-regulated in the right sample vs. the left sample, green genes are down-regulated in the right sample vs. the left sample, and blue genes are not differentially expressed. OA_Lc, OA_Sk, OA_Cs, OA_Af were representive of O. asiaticus individuals feeding L. chinensis, S. krylovii, C. squarrosa, A. frigida, respectively. (TIF) [file pone.0186397.s005.tif]
