# Supplementary material for: Diet alters performance and transcription patterns in Oedaleus asiaticus (Orthoptera: Acrididae) grasshoppers
Source: PLoS One. 2017 Oct 12;12(10):e0186397. doi: 10.1371/journal.pone.0186397 (PMC5638516; doi:10.1371/journal.pone.0186397)
Supplement: S1 Table — (DOCX) [file pone.0186397.s006.docx]

**S1Table.** Designed sequences of qRT-PCR primers for ten candidate genes

| Candidate gene | Sequence of primers for real-time PCR (5' to 3') | |
| --- | --- | --- |
| *CHS* | Forward | GGGTACAGCTCTGCAGCTCG |
|  | Reverse | GGTGGAAGGCAGGCAGCAAT |
| *CUP*2 | Forward | GTGAATCGCTCGTAATTTCTGT |
|  | Reverse | ACATCTTATGTATGGGTCTTCT |
| *LCP* | Forward | AGTGTACTGAGCGCTCTGG |
|  | Reverse | GTCTCCATCTCTTCGCAGT |
| *SEG* | Forward  Reverse | ATCCCGATCCAGAACCAGATGC  AAGACGATGCCGACAAAGAGCC |
| *CUP*1 | Forward  Reverse | TGAGATGGCACCAGATTATGAC  ATACTCTTGAGTATGGCGATTT |
| *CAT* | Forward Reverse | ACAGTGGCATCTCGGATG  TCAGTCGGCGATGTGGAG |
| *CYP* | Forward Reverse | CCTTCTTGCTGGCTATGAAAC  CCACATCAATGGTCACTTCTG |
| *HSP* | Forward Reverse | TCAAGCATTGCGGCAGCCTA  GTGAACTGCTGGTGCCCGAT |
| *SBD* | Forward Reverse | GTAGACGGACGAAGGCGGTG  GGCGAAACGTTTGACCTGGC |
| *NSO* | Forward Reverse | CTTGTCTTGGCCGACAGGGG  CATCGGGCTTGCAGTGAGGT |
| *β-actin* | Forward Reverse | CCCATCTATGAAGGTTACGC  CTTGATGTCACGGACGATTT |
