# Supplementary material for: Diet alters performance and transcription patterns in Oedaleus asiaticus (Orthoptera: Acrididae) grasshoppers
Source: PLoS One. 2017 Oct 12;12(10):e0186397. doi: 10.1371/journal.pone.0186397 (PMC5638516; doi:10.1371/journal.pone.0186397)
Supplement: S2 Table — (DOCX) [file pone.0186397.s007.docx]

**S2 Table.** *O. asiaticus* survival rate from 3^rd^ to 5^th^ instar ± SE, mean dry mass (mg ±SE) of 5^th^ instar nymphs, mean developmental time (days± SE) from 3^rd^ instar to 5^th^ instar, growth rate (mg/day ±SE) and overall performance (survival rate (SR) × growth rate (GR) ±SE) when fed on Lc (*L. chinensis*), Sk (*S. krylovii*), Cs (*C. squarrosa*) and Af (*A. frigida*).

| Food plant | Survival rate (%) | Dry mass  (mg) | Developmental time (days) | Growth rate  (mg/day) | Overall performance |
| --- | --- | --- | --- | --- | --- |
| *L. chinensis* | 75.8±5.53 | 218.2±20.7 | 21.2±2.2 | 10.3±1.2 | 7.8±1.7 |
| *S. krylovii* | 78.8±6.72 | 238.7±19.6 | 19.2±3.4 | 12.4±1.4 | 9.8±2.6 |
| *C. squarrosa* | 72.6±8.65 | 246.7±11.4 | 21.8±1.9 | 11.3±1.0 | 8.2±2.1 |
| *A. frigida* | 45.6±5.76 | 168.6±17.2 | 28.3±2.6 | 6.0±1.5 | 2.7±1.3 |
