# Supplementary material for: Diet alters performance and transcription patterns in Oedaleus asiaticus (Orthoptera: Acrididae) grasshoppers
Source: PLoS One. 2017 Oct 12;12(10):e0186397. doi: 10.1371/journal.pone.0186397 (PMC5638516; doi:10.1371/journal.pone.0186397)
Supplement: S3 Table — (DOCX) [file pone.0186397.s008.docx]

**S3 Table.** Annotation results of unigenes

|  | Number of Unigenes | Percentage (%) |
| --- | --- | --- |
| Annotated in NR | 33847 | 19.7 |
| Annotated in NT | 6155 | 3.58 |
| Annotated in KEGG | 7571 | 4.4 |
| Annotated in SwissProt | 16759 | 9.75 |
| Annotated in GO | 28324 | 16.49 |
| Annotated in KOG | 11700 | 6.81 |
| Annotated in all Databases | 2633 | 1.53 |
| Annotated in at least one Database | 45517 | 26.5 |
| Total Unigenes | 171743 | 100 |
