# Supplementary material for: Diet alters performance and transcription patterns in Oedaleus asiaticus (Orthoptera: Acrididae) grasshoppers
Source: PLoS One. 2017 Oct 12;12(10):e0186397. doi: 10.1371/journal.pone.0186397 (PMC5638516; doi:10.1371/journal.pone.0186397)
Supplement: S4 Table — (DOCX) [file pone.0186397.s009.docx]

**S4 Table.** The same differentially expressed genes (qvalue <0.05, |log2.Fold_change|>1, only annotated and down-regulated genes) of *O. asiaticus* feeding *A. frigida* compared with individuals feeding the other three plants *L. chinensis*, *S. krylovii*, *C. squarrosa*.

| Summarized Terms | Gene id | Nr annotation | log2.Fold_change  OA_Af vs OA_Cs | log2.Fold_change  OA_Af vs OA_Lc | log2.Fold_change  OA_Af vs OA_Sk |
| --- | --- | --- | --- | --- | --- |
| Insect cuticle biosynthesis related | c84444_g1 | Endocuticle structural glycoprotein SgAbd-9, partial [Zootermopsis nevadensis] | -1.6707 | -1.9205 | -1.8287 |
|  | c73001_g2 | cuticular protein RR-1 motif 8 [Antheraea yamamai] | -2.0254 | -2.2295 | -2.0847 |
|  | c84969_g3 | PREDICTED: endocuticle structural glycoprotein ABD-4 [Ceratitis capitata | -1.3228 | -2.4114 | -1.56 |
|  | c73896_g1 | Cysteine-rich with EGF-like domain protein 2 [Zootermopsis nevadensis] | -1.3303 | -1.6483 | -1.5698 |
|  | c78558_g5 | RecName: Full=Cuticle protein 1; AltName: Full=Bc-NCP1 [Blaberus craniifer] | -2.8168 | -3.9366 | -3.4249 |
|  | c89119_g1 | TPAputative cuticle protein [Danaus plexippus] | -2.2912 | -2.688 | -2.0989 |
|  | c69967_g1 | RecName: Full=Endocuticle structural glycoprotein SgAbd-5 [Schistocerca gregaria] | -1.9317 | -2.241 | -2.2657 |
|  | c84435_g1 | RecName: Full=Endocuticle structural glycoprotein SgAbd-3 [Schistocerca gregaria] | -1.3249 | -1.5913 | -1.6951 |
|  | c83044_g2 | Collagen alpha-1(XI) chain, partial [Zootermopsis nevadensis] | -1.5252 | -1.8896 | -1.7217 |
|  | c82379_g1 | PREDICTED: endocuticle structural protein SgAbd-6 [Ceratitis capitata] | -1.891 | -2.142 | -2.1144 |
|  | c87369_g1 | RecName: Full=Cuticle protein 2; AltName: Full=LM-ACP 2 [Locusta migratoria] | -2.2219 | -2.0081 | -2.1354 |
|  | c83430_g2 | RecName: Full=Endocuticle structural glycoprotein SgAbd-1 [Schistocerca gregaria] | -5.0989 | -6.9803 | -5.7492 |
|  | c74226_g1 | chitin synthase 1 variant B [Locusta migratoria manilensis] | -1.6108 | -1.7995 | -1.7137 |
|  | c84112_g1 | larvae cuticle protein [Choristoneura fumiferana] | -1.5159 | -1.9608 | -1.9977 |
|  | c83044_g1 | PREDICTED: collagen alpha-1(XI) chain-like [Harpegnathos saltator] | -1.6374 | -1.8678 | -1.5292 |
|  | c84240_g1 | Outer dense fiber protein 3 [Zootermopsis nevadensis] | -1.7881 | -1.8869 | -1.996 |
|  | c70420_g1 | hypothetical protein YQE_04817, partial [Dendroctonus ponderosae] | -1.5161 | -1.7447 | -1.7555 |
| DNA replication related | c86763_g1 | DNA mismatch repair protein Msh6 [Zootermopsis nevadensis] DNA | -1.3793 | -1.5271 | -1.5706 |
|  | c64439_g1 | DNA primase large subunit [Zootermopsis nevadensis] | -1.6157 | -1.8769 | -1.8812 |
|  | c88149_g2 | Transposable element Tcb2 transposase, partial [Stegodyphus mimosarum] | -1.7208 | -1.8248 | -1.95 |
|  | c77582_g1 | thymidylate synthase [Litopenaeus vannamei] | -1.5246 | -1.7483 | -1.9018 |
|  | c70950_g1 | Deoxyuridine 5'-triphosphate nucleo tido hydrolase [Zootermopsis nevadensis] | -2.1559 | -1.7672 | -2.2198 |
|  | c79027_g1 | PREDICTED: DNA (cytosine-5)-methyltransferase PliMCI-like [Megachile rotundata] | -1.2912 | -1.6523 | -1.7724 |
|  | c80389_g1 | endonuclease-reverse transcriptase [Eyprepocnemis plorans plorans] | -1.7319 | -2.0619 | -2.0095 |
|  | c80451_g1 | DNA polymerase alpha catalytic subunit, putative [Pediculus humanus corporis]>gi\|212509481\|gb\|EEB12850.1\| | -2.5729 | -2.4524 | -2.6765 |
|  | c78893_g2 | PREDICTED: DNA polymerase epsilon catalytic subunit A [Bombyx mori] | -1.2479 | -1.6859 | -1.4146 |
|  | c66549_g1 | Histone H2A [Zootermopsis nevadensis] | -1.599 | -1.8744 | -1.644 |
|  | c80333_g1 | Ribonucleoside-diphosphate reductase subunit M2 [Zootermopsis nevadensis] | -3.041 | -3.2137 | -4.3685 |
|  | c86889_g2 | Mcm5 [Locusta migratoria] | -1.1971 | -1.5708 | -1.212 |
|  | c78788_g1 | Mcm4 protein [Locusta migratoria] | -1.5983 | -1.7347 | -1.9043 |
|  | c79035_g2 | hypothetical protein TcasGA2_TC005282 [Tribolium castaneum] | -1.4242 | -2.0516 | -2.1683 |
|  | c72384_g1 | Mcm2 [Locusta migratoria] | -1.6705 | -2.4971 | -2.6318 |
| Biosynthesis and metabolism of carbohydrate, fat and protein related | c85042_g5 | glucosyl glucuronosyl transferases [Locusta migratoria] | -1.3418 | -1.6651 | -1.5631 |
|  | c75531_g2 | Dolichyl-diphosphooligosaccharide--protein glycosyltransferase subunit 2 [Zootermopsis nevadensis] | -3.9371 | -4.5939 | -5.2497 |
|  | c81215_g3 | Alcohol dehydrogenase [NADP+] [Zootermopsis nevadensis] | -2.1109 | -2.1361 | -2.0601 |
|  | c82327_g3 | Dolichyl pyrophosphate Man9GlcNAc2 alpha-1,3-glucosyltransferase, partial [Zootermopsis nevadensis] | -1.7038 | -1.5802 | -1.7054 |
|  | c67285_g1 | glucosyl glucuronosyl transferases [Locusta migratoria] | -1.4871 | -1.7251 | -1.5045 |
|  | c82932_g1 | hypothetical protein KGM_19483 [Danaus plexippus] | -2.0789 | -2.1382 | -2.3661 |
|  | c78115_g1 | Dolichyl-diphosphooligosaccharide--protein glycosyl transferase 48 kDa subunit [Zootermopsis nevadensis] | -2.4823 | -2.6514 | -2.9965 |
|  | c88249_g1 | PREDICTED: 6-phosphogluconate dehydrogenase, decarboxylating isoform X1 [Strongylocentrotus purpuratus]>gi\|390352586\| | -1.3342 | -1.5551 | -1.4872 |
|  | c79822_g1 | Dolichyl-diphosphooligosaccharide--protein glycosyl transferase subunit STT3A [Zootermopsis nevadensis] | -1.7202 | -1.9017 | -1.8119 |
|  | c84708_g1 | PREDICTED: LOW QUALITY PROTEIN: glucose dehydrogenase [Bombus impatiens] | -1.9036 | -1.7999 | -1.8413 |
|  | c81743_g2 | PREDICTED: L-aminoadipate-semialdehyde dehydrogenase-like [Crassostrea gigas]>gi\|762156683\|ref\|XP_011416476.1\| | -1.8604 | -1.8745 | -2.8806 |
|  | c85926_g1 | GalNAc:polypeptide N-acetyl galactosaminyl transferase, putative [Pediculus humanus corporis] | -2.0146 | -2.1095 | -1.753 |
|  | c76697_g1 | Oligosaccharyltransferase complex subunit ostc-B [Zootermopsis nevadensis] | -1.5741 | -2.038 | -1.747 |
|  | c66378_g1 | Macrophage mannose receptor 1, partial [Harpegnathos saltator] | -1.4534 | -1.7468 | -1.5998 |
|  | c83276_g1 | glucuronosyltransferase, partial [Zootermopsis nevadensis] | -2.0806 | -2.625 | -2.6558 |
|  | c85750_g3 | N-acetylglucosamine pyrophosphorylases 1 [Locusta migratoria] | -1.6 | -1.895 | -1.7456 |
|  | c85204_g1 | PREDICTED: venom dipeptidyl peptidase 4 isoform X2 [Cerapachys biroi] | -1.3772 | -1.6334 | -1.6791 |
|  | c81572_g2 | Histone-lysine N-methyltransferase SETMAR, partial [Stegodyphus mimosarum] | -1.4508 | -2.1054 | -2.3242 |
|  | c84670_g1 | PREDICTED: D-aspartate oxidase [Pogonomyrmex barbatus] | -1.474 | -1.5462 | -1.5147 |
|  | c83848_g1 | Golgi integral membrane protein 4 [Zootermopsis nevadensis] | -2.4266 | -2.1098 | -1.9905 |
|  | c79014_g1 | Heterogeneous nuclear ribonucleoprotein H [Zootermopsis nevadensis] | -1.4213 | -1.5699 | -1.3785 |
|  | c82265_g1 | peptidyl-prolyl isomerase-1 [Locusta migratoria] | -1.9385 | -2.3789 | -2.1791 |
|  | c88896_g1 | PREDICTED: zinc finger BED domain-containing protein 5-like [Microplitis demolitor] | -1.2839 | -1.6841 | -1.5134 |
|  | c75635_g1 | protein disulfide-isomerase [Schistocerca gregaria] | -1.7331 | -2.1309 | -3.0563 |
|  | c83554_g5 | Protein disulfide-isomerase A5 [Zootermopsis nevadensis] | -1.7759 | -2.7787 | -2.9817 |
|  | c85926_g1 | UDP-GalNAc:polypeptide N-acetyl galactosaminyl transferase, putative [Pediculus humanus corporis] | -1.6791 | -2.1402 | -2.1862 |
|  | c79430_g1 | Heterogeneous nuclear ribonucleoprotein K [Zootermopsis nevadensis] | -3.4842 | -4.3708 | -4.1368 |
|  | c87595_g1 | PREDICTED: E3 ubiquitin-protein ligase UHRF1-like [Saccoglossus kowalevskii] | -2.0784 | -2.0358 | -2.2102 |
|  | c85750_g3 | UDP N-acetylglucosamine pyrophosphorylases 1 [Locusta migratoria] | -1.9372 | -1.6644 | -1.6308 |
|  | c76783_g1 | ER protein gp78 [Locusta migratoria] | -3.5566 | -3.2289 | -3.9387 |
|  | c86327_g1 | Microsomal triglyceride transfer protein large subunit [Zootermopsis nevadensis] | -1.5176 | -1.7556 | -1.7748 |
|  | c80133_g1 | Lipoyltransferase 1, mitochondrial [Zootermopsis nevadensis] | -2.0669 | -2.4696 | -2.5855 |
|  | c85835_g2 | Putative fatty acyl-CoA reductase [Zootermopsis nevadensis] | -1.795 | -1.8851 | -1.2809 |
|  | c82584_g1 | Carnitine O-palmitoyltransferase 1, liver isoform [Zootermopsis nevadensis] | -1.7254 | -1.9842 | -1.926 |
|  | c81249_g3 | Myelin expression factor 2 [Zootermopsis nevadensis] | -1.6945 | -2.273 | -2.0949 |
|  | c86921_g3 | hexamerin-like protein 1 [Locusta migratoria] | -2.9539 | -3.2423 | -3.097 |
| Others related | c72762_g3 | GTP:AMP phosphotransferase mitochondrial [Zootermopsis nevadensis] | -1.6474 | -1.6336 | -1.8008 |
|  | c70647_g1 | type I signal peptidase 21 kDa subunit [Locusta migratoria manilensis] | -1.8807 | -2.1676 | -2.2884 |
|  | c78589_g1 | Hypoxia up-regulated protein 1 [Zootermopsis nevadensis] | -2.8229 | -2.7893 | -4.2044 |
|  | c88121_g1 | Putative odorant-binding protein A10 OS=Drosophila melanogaster GN=a10 PE=1 SV=2 | -1.3607 | -1.911 | -2.1237 |
|  | c73744_g1 | PREDICTED: angiopoietin-4 [Tribolium castaneum] | -2.9484 | -3.2131 | -5.1532 |
|  | c79918_g1 | PREDICTED: prostaglandin reductase 1-like [Nasonia vitripennis]>gi\|645005875\|ref\|XP_ 008204228.1\| | -1.9729 | -2.1725 | -2.3637 |
|  | c54528_g1 | translocon-associated protein subunit delta-like (TRAP-delta) [Coptotermes formosanus] | -1.6707 | -1.9205 | -1.8287 |
|  | c83837_g2 | Juvenile hormone epoxide hydrolase 1 [Zootermopsis nevadensis] | -1.4508 | -2.1054 | -2.3242 |
