# Supplementary material for: Diet alters performance and transcription patterns in Oedaleus asiaticus (Orthoptera: Acrididae) grasshoppers
Source: PLoS One. 2017 Oct 12;12(10):e0186397. doi: 10.1371/journal.pone.0186397 (PMC5638516; doi:10.1371/journal.pone.0186397)
Supplement: S5 Table — (DOCX) [file pone.0186397.s010.docx]

**S5 Table.** The same differentially expressed genes (qvalue <0.05, |log2.Fold_change|>1, only annotated and up-regulated genes) of *O. asiaticus* feeding *A. frigida* compared with individuals feeding the other three plants *L. chinensis*, *S. krylovii*, *C. squarrosa*.

| Gene id | Nr annotation | log2.Fold_change  OA_Af vs OA_Cs | log2.Fold_change  OA_Af vs OA_Lc | log2.Fold_change  OA_Af vs OA_Sk |
| --- | --- | --- | --- | --- |
| c88585_g1 | heat shock protein 19.8 [Oxya chinensis] | 2.6398 | 1.7942 | 1.2044 |
| c82555_g1 | carboxylesterase [Oxya chinensis] | 2.4508 | 2.6846 | 2.1358 |
| c87127_g1 | PREDICTED: inositol oxygenase isoform X1 [Athalia rosae] | 1.4662 | 1.7369 | 1.7716 |
| c87438_g1 | RecName: Full=Cytochrome P450 6k1; AltName: Full=CYPVIK1 [Blattella germanica] | 3.0982 | 4.2497 | 2.9649 |
| c82717_g4 | gastric caeca sugar transporter [Locusta migratoria] | 2.8561 | 4.6831 | 3.2754 |
| c87294_g1 | PREDICTED: inter-alpha-trypsin inhibitor heavy chain H3 isoform X2 [Acyrthosiphon pisum] | 2.2103 | 2.64 | 1.9598 |
| c82717_g3 | gastric caeca sugar transporter [Locusta migratoria] | 2.1937 | 1.9203 | 1.476 |
| c86816_g2 | carboxylesterase [Locusta migratoria] | 5.545 | 6.7399 | 4.1891 |
| c75889_g1 | phosphoserine transaminase [Blattella germanica] | 1.775 | 2.4851 | 1.5368 |
| c80735_g3 | Sorbitol dehydrogenase [Zootermopsis nevadensis] | 1.5092 | 2.1941 | 2.0727 |
| c70968_g1 | Hexokinase type 2 [Zootermopsis nevadensis] | 1.9478 | 2.3036 | 2.8136 |
| c85322_g1 | PREDICTED: putative inorganic phosphate cotransporter isoform X1 [Megachile rotundata] | 2.4787 | 3.0847 | 1.7357 |
| c78438_g2 | pacifastin-related peptide precursor PP-5 [Schistocerca gregaria] | 2.5088 | 3.2025 | 2.4777 |
| c76294_g2 | SCAN domain-containing protein 3 [Larimichthys crocea]>gi\|808863429\|gb\|KKF14764.1\| | 1.8533 | 2.3521 | 2.1856 |
| c85540_g2 | PREDICTED: serine hydroxymethyltransferase, cytosolic [Monomorium pharaonis] > gi \|826419753\|ref\|XP_012524775.1\| | 3.4908 | 2.1705 | 2.9526 |
| c85487_g1 | PREDICTED: nose resistant to fluoxetine protein 6-like [Fopius arisanus] | 1.3783 | 1.6137 | 1.4707 |
| c85215_g1 | PREDICTED: inter-alpha-trypsin inhibitor heavy chain H4-like isoform X2 [Bombyx mori] | 3.372 | 3.5178 | 3.5658 |
| c89091_g1 | Insulin receptor [Zootermopsis nevadensis] | 2.2158 | 2.2177 | 2.9995 |
| c83962_g1 | PREDICTED: LOW QUALITY PROTEIN: putative leucine-rich repeat-containing protein DDB_G0290503 [Tribolium castaneum] | 1.5628 | 2.0098 | 1.2704 |
| c71543_g1 | PREDICTED: uncharacterized protein LOC105383056 [Plutella xylostella] | 1.3755 | 3.1978 | 2.7102 |
| c77777_g2 | hypothetical protein TcasGA2_TC004196 [Tribolium castaneum] | 2.3703 | 2.575 | 2.1601 |
| c86628_g1 | hypothetical protein DAPPUDRAFT_51498 [Daphnia pulex] | 1.818 | 1.8427 | 1.6281 |
| c88979_g4 | hypothetical protein L798_10809 [Zootermopsis nevadensis] | 2.2933 | 3.7515 | 5.6534 |
| c78178_g1 | Xaa-Pro amino peptidase 2 [Zootermopsis nevadensis] | 1.7146 | 2.9979 | 2.4449 |
| c80796_g3 | AAEL012443-PA [Aedes aegypti]>gi\|108871163\|gb\|EAT35388.1\| AAEL012443-PA [Aedes aegypti] | 2.4013 | 2.145 | 2.8095 |
| c89356_g2 | hypothetical protein TcasGA2_TC004196 [Tribolium castaneum] | 2.9751 | 3.7466 | 2.8491 |
| c84769_g2 | Leucine-rich repeat-containing protein 20 [Zootermopsis nevadensis] | 1.6881 | 1.9271 | 1.8849 |
| c67502_g1 | 4-hydroxyphenylpyruvate dioxygenase [Zootermopsis nevadensis] | 1.4104 | 1.6001 | 1.4473 |
| c73696_g1 | PREDICTED: putative protein FAM200B-like isoform X1 [Python bivittatus]>gi\|  602668075\|ref\|XP_007439591.1\| | 2.0322 | 1.7679 | 1.5262 |
| c68127_g1 | Homogentisate 1,2-dioxygenase [Zootermopsis nevadensis] | 2.1929 | 2.243 | 2.3718 |
